# Global source-sink dynamics of dengue viruses and epidemic establishment in areas on the fringe of endemic transmission

**DOI:** 10.1093/nsr/nwag372

**Published:** 2026-06-15

**Authors:** Zhiyuan Chen, Marta Giovanetti, Zachary J Madewell, Andi Sun, Wenzhi Li, Chunmin Li, Haoyin Yu, Jimin Sun, Liyun Jiang, Pengzhe Qin, Xinwei Wu, Marco Ajelli, Hongjie Yu

**Affiliations:** School of Public Health, Key Laboratory of Public Health Safety, Ministry of Education, Fudan University, Shanghai 200032, China; Department of Science and Bio-Technology, Università Campus Bio-Medico di Roma, Rome 00128, Italy; Oswaldo Cruz Institute, Oswaldo Cruz Foundation, Rio de Janeiro 21040-900, Brazil; Division of Vector-Borne Diseases, Centers for Disease Control and Prevention, San Juan 00920, Puerto Rico; School of Public Health, Key Laboratory of Public Health Safety, Ministry of Education, Fudan University, Shanghai 200032, China; School of Public Health, Key Laboratory of Public Health Safety, Ministry of Education, Fudan University, Shanghai 200032, China; Yunnan Institute of Parasitic Diseases, Yunnan Provincial Key Laboratory of Vector-borne Disease Control and Research, Yunnan International Joint Laboratory of Tropical Infectious Diseases, Kunming 650500, China; Yunnan Institute of Parasitic Diseases, Yunnan Provincial Key Laboratory of Vector-borne Disease Control and Research, Yunnan International Joint Laboratory of Tropical Infectious Diseases, Kunming 650500, China; Zhejiang Provincial Center for Disease Control and Prevention, Hangzhou 310051, China; Guangzhou Center for Disease Control and Prevention (Guangzhou Health Supervision institute), Guangzhou 510440, China; Guangzhou Center for Disease Control and Prevention (Guangzhou Health Supervision institute), Guangzhou 510440, China; Guangzhou Center for Disease Control and Prevention (Guangzhou Health Supervision institute), Guangzhou 510440, China; Laboratory for Computational Epidemiology and Public Health, Department of Epidemiology and Biostatistics, Indiana University School of Public Health-Bloomington, Bloomington, IN 47405, USA; School of Public Health, Key Laboratory of Public Health Safety, Ministry of Education, Fudan University, Shanghai 200032, China; Shanghai Institute of Infectious Disease and Biosecurity, Fudan University, Shanghai 200032, China; Department of Infectious Diseases, Huashan Hospital, Fudan University, Shanghai 200040, China

**Keywords:** dengue virus, endemic area, fringe area, source-sink model, dispersal dynamic

## Abstract

The global expansion of dengue virus (DENV) poses a major public health threat, yet its circulation dynamics remain poorly understood. By integrating global genetic, epidemiological, and mobility data within a phylodynamic framework, we show that DENV circulation follows a source-sink model, with tropical endemic regions acting as persistent viral reservoirs (‘sources’) that seed epidemics in temperate areas (‘sinks’). Most areas at the fringe of endemic transmission exhibit sink-like characteristics with exception of South China. We identify a low-intensity dispersal route from South China to endemic Asia compared with the high-intensity source-to-sink direction, along with occasional DENV1 overwintering events in South China. Together, these findings suggest that South China occupies an intermediate state between endemic and non-endemic areas. These global DENV pathways were primarily driven by air travel and were reshaped by COVID-19 pandemic-related perturbations. Our findings highlight that dengue circulates within a fully interconnected global system that requires a shift from localized control to globally coordinated intervention efforts, as well as emphasize the importance of year-round genomic surveillance at the human-vector interface in fringe areas.

## INTRODUCTION

Dengue virus (DENV) is an arbovirus that can be transmitted between humans via the bites of *Aedes aegypti* and *Ae. albopictus* within a widespread urban transmission cycle. In recent decades, dengue has increased dramatically [1], causing an estimated 100–400 million infections annually worldwide [2]. The recent expansion of DENV has been associated with expansion and enhancement of mosquito suitability driven by climate and increasing human-vector interactions arising from urbanization and human mobility [3–8]. With the lengthening of the mosquito season, DENV infections have increasingly established epidemic cycles in the southern parts of the United States [9], Europe [10], and China [11]. As such, there is justifiable concern that areas on the fringe of endemic DENV transmission may be at risk of becoming new endemic regions [2,4,12].

Although DENV established endemic transmission in urban cycles several hundred years ago [13], highly effective therapeutics are still lacking and vaccines are not widely adopted [14]. One of the key challenges in DENV vaccine development is the need for a tetravalent formulation that induces simultaneous and balanced protection against all four serotypes [15], especially given the severe risk associated with secondary infections by a heterotypic serotype primarily due to antibody-dependent enhancement. As a result, vector control measures still remain critical for reducing the annual global burden of dengue [15,16], while prevention strategies based on vector control may be challenging and hard to sustain [17], especially

in the presence of the frequent reintroduction of viremic travelers [18].

The challenge of frequent viral reintroductions highlights the limitations of localized control and underscores the critical need to understand global DENV dispersal and maintenance dynamics, since reintroduction from distant sources can undermine local vector control strategies. Phylodynamic approaches are powerful tools to accomplish this goal by providing insights into the evolution and transmission dynamics of pathogens using genomic data [19]. This approach was applied for other mosquito-borne diseases such as Zika [20] and yellow fever [21]. While such methods have been used to explore the global and regional dispersal patterns of individual DENV serotypes [6,22–25], key knowledge gaps still remain. Specifically, the processes governing the annual establishment of dengue in areas on the fringe of endemic transmission, their potential trends toward endemicity, and the impact of major global disruptions like the COVID-19 pandemic have not been fully understood. Additionally, recent increases in genomic data availability, coupled with advances in phylodynamic methodology (for example, incorporating travel history [26]), have enabled us to refine and extend our understanding of underexplored regions (for example, Africa).

In this study, we integrated genetic, epidemiological, and mobility data into a phylodynamic framework to investigate the global spatiotemporal dispersal and persistence dynamics of DENVs from 2010 to 2025. Specifically, our study aims to elucidate global DENV circulation patterns, characterize its expansion toward fringe areas, and assess their trends toward endemicity. We placed particular emphasis on viral transitions between endemic regions and areas at the current fringe of endemicity, as well as on the annual establishment of epidemics in fringe area. Together, these insights provide an evidence base to inform the design of globally coordinated control strategies.

## RESULTS

### Distinct seasonality patterns between endemic and fringe areas

**Figure 1.**
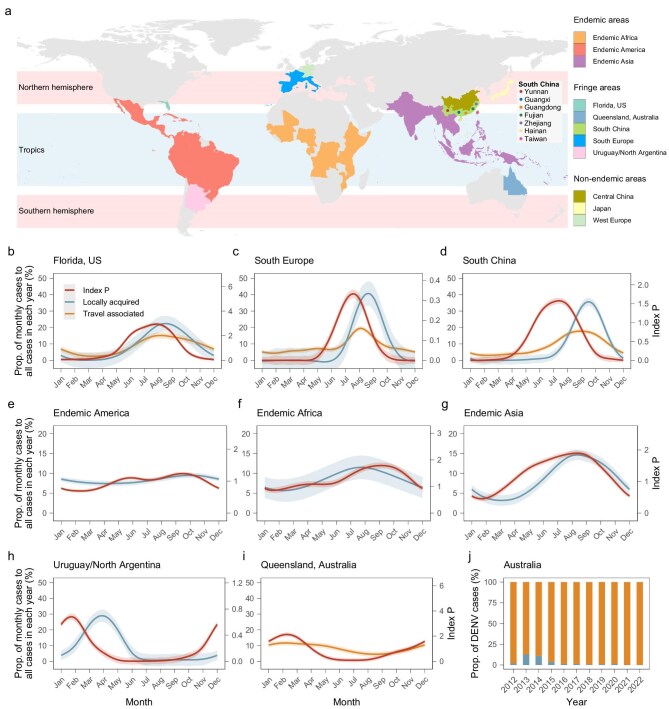
Seasonality of dengue epidemics by travel status and index P across defined geographic locations. (a) The spatial ranges of three endemic areas, five fringe areas, and three non-endemic areas we defined in this study. Colored points in South China represent provinces that were classified as Category I areas (highest risk of dengue transmission) by National Disease Control and Prevention Administration and accounted for the majority of locally-acquired dengue cases in China. The light-blue and light-red shaded areas represent tropical areas and the two hemispheres adjacent to tropical areas, respectively. (b–i) Mean seasonality curve of dengue epidemics stratified by travel status and index P for each geographic location from 2010 to 2025. All country-specific curves by each year were presented in Fig. S3. While the seasonality varies across regions, it is important to stress that in endemic areas, the resulting average behavior is the result of different seasonal trends as it is the case in Africa with a clear peak of index P around July–September driven by countries in West Africa. The shaded area around the fitted curve represents the 95% confidence interval. As most dengue cases reported in Australia were travel-associated cases (see panel j), a potential association with the release of Wolbachia-infected mosquitos [30], we used the curve of all reported dengue cases as the travel-associated curve because the data did not distinguish travel-associated from locally acquired cases. DENV, dengue virus. Review drawing number: GS 京(2026)1788号.

Based on the boundaries of endemicity [27,28], epidemic characteristics, and data availability, we categorized the world into three endemic areas (endemic America, endemic Africa, and endemic Asia), three non-endemic areas (Central China, Japan, and West Europe), and five areas at the fringe of endemicity (Florida, South Europe, South China, Uruguay/North Argentina, and Queensland) (Fig. 1a, Tables S1 and S2). We define fringe areas as regions situated at the epidemiological interface of endemicity, characterized by a spectrum of unstable transmission ranging from localized seasonal outbreaks to emerging year-round circulation that lacks a history of established year-to-year persistence characteristic of endemic reservoirs (Figs S1, S2 and Table S3), with quantitative definition in Methods section. For each location, we assembled a dengue surveillance dataset from 2010 to 2025 that includes information on monthly reported cases disaggregated by travel status (locally acquired or travel-associated).

The epidemic peaks of locally acquired dengue cases in the USA (Florida), South Europe, and South China occurred around August–September, whereas peaks of travel-associated cases were ∼1 month earlier and with a long tail toward the first half of year (Fig. 1b–d). The continuous introductions of imported cases in the spring have not triggered the emergence of local outbreaks, which might be due to unsuitable climate conditions and limited mosquito density. Specifically, the values of index P (climate-driven transmission potential of DENV transmitted by *Ae. aegypti* [29]) remained relatively low in the spring and showed a similar temporal pattern to that of local dengue cases with a time lag of around 1–2 months (Fig. 1b–d). Patterns in Uruguay/North Argentina also showed a 2-month lag between index P and local dengue cases, peaking around the end of January and March, respectively (Fig. 1h). In Australia, locally acquired DENV infections were rarely reported from 2012 to 2022 [30], despite the introduction of imported dengue cases occurring each month (Fig. 1i, j).

In comparison to the seasonality in fringe areas, the temporal patterns of local cases and index P vary across years and countries within each endemic area (Fig. S3). Generally, they were less seasonal in endemic regions, in which local acquired cases can be detected and average of index P can be maintained at a certain level (>0.5, higher index P is one indicator of favorable climatic conditions for DENV circulation [29]) throughout the whole year (Fig. 1e–g).

### Three modes of global DENV dispersal

**Figure 2.**
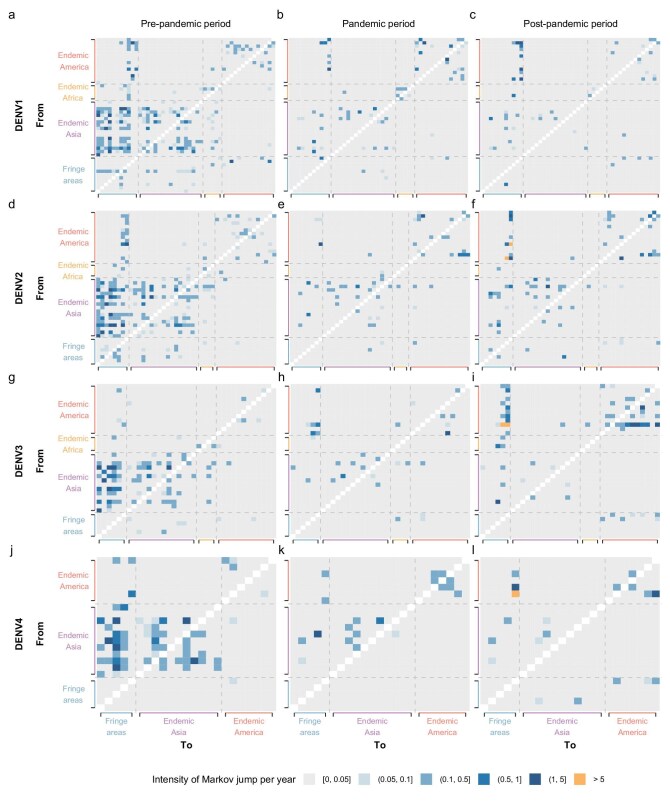
Global migration dynamics of four DENV serotypes through periods at the sub-location level. (a–l) Estimates of the number of location transition events (Markov jump) per year between each pair of geographic sub-locations during the pre-pandemic, pandemic, and post-pandemic periods. Analyses are based on the posterior summaries of the Markov jumps under a time-inhomogeneous GLM-diffusion phylogeographic model with only air traffic data as the predictor of relative transition rates. The names of sub-locations are not shown in the axis, but are instead categorized within endemic America, endemic Africa, endemic Asia, and fringe areas; the full graphs with axis text information were presented in Figs S7–S10. The results were run using data selected by infection-informed sub-sampling scheme, while another output based on spatially even sub-sampling scheme was presented in Fig. S11.

By integrating a globally curated genetic dataset (Figs S4–S6) and case travel history data (Tables S4 and S5), we reconstructed the global dispersal patterns of DENVs from 2010 to 2025 at three spatial resolutions: sub-location, sub-region, and regional level (Fig. S2). As a baseline (2010–2019, prior to the COVID-19 pandemic), our results identified three major dispersion modes at the sub-location level: (i) internal dispersal within each endemic area; (ii) dispersal from endemic areas to those areas on the fringe of endemic transmission; and (iii) low-intensity spread from fringe areas to endemic areas (Fig. 2a, d, g, j; Figs S7–S10).

**Figure 3.**
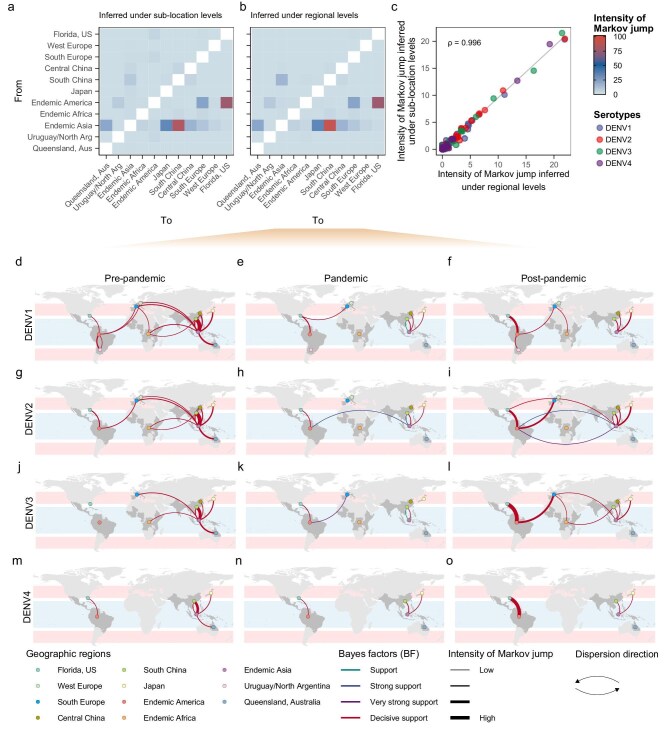
Global migration routes of DENV through three periods at the regional level. (a) The migration matrix of DENV (four DENV serotypes combined) inferred under the sub-location level. The sub-location results are aggregated at the region scale for direct comparison. (b) The migration matrix of DENV inferred under the regional level. (c) Association between the serotype- and epoch-specific vectorized transition matrices between each pair of regions, including the Pearson correlation coefficient. (d–o) Regional-level analyses: estimates of the annual intensity of Markov jump events (migration events) for each DENV serotype between each pair of geographic locations during the pre-pandemic (2010–2019), pandemic (2020–2021), and post-pandemic (2022–2025) periods, respectively. The colored lines represent statistical support for a given viral migration route using Bayes factor (BF), where only migration routes with a BF ≥ 3 are shown. The thicknesses of the curves refer to the annual intensity of Markov jump, where only migration routes with an intensity ≥0.5 are shown. The orientation of the curve (convex vs. concave) indicates the directionality of the dispersion (always counter-clockwise). The light-blue and light-red shaded areas represent tropical regions and the two hemispheres adjacent to tropical areas, respectively. This analysis was performed using genetic datasets selected from infection-informed sub-sampling scheme, while another output based on spatially even sub-sampling scheme was presented in Fig. S12. Review drawing number: GS 京(2026)1788号.

In the first mode, average annual intensity of between-sublocation DENV spread within endemic Asia (0.065 for all four serotypes) is four times that of endemic America (0.016) and twice that of Africa (0.032) (Fig. 2a, d, g, j); these results are robust to the sub-sampling schemes (Fig. S11). However, within each endemic area, outward diffusion intensity varied across each country, with instances showing limited virus movement (Fig. 2). This is consistent with a previous study [31] and might be attributed to founder effect and variations in country-specific vector control efforts and population susceptibility, as well as the varied availability of genetic data. Furthermore, we rarely observed between-region movements among endemic areas, except for spread from endemic Asia to endemic Africa, which suggest the relatively independent maintenance of virus ecology within each endemic area. These results were further validated at the region-level analyses, in which we only observed a decisive-supported [Bayes factor (BF) ≥ 1000] dispersal route from endemic Asia to Africa for DENV1, DENV2, and DENV3 (Fig. 3d, g, j).

The regional-level analyses exhibited a highly correlated result with the analyses at the sub-location level (ρ = 0.996, Fig. 3a–c). At the regional level, the second mode was primarily characterized by the spread from endemic areas to fringe and non-endemic areas, especially the routes from endemic America to Florida, US, as well as from endemic Asia to the Europe and Western Pacific region (China, Queensland, and Japan) across all four serotypes (Fig. 3d, g, j, m; robust to subsampling scheme: Figs S12–S14). Decisive support (BF ≥ 1000) for DENV1 spread from endemic Africa to Europe was also observed (Fig. 3d), as well as DENV1 and DENV2 spread from endemic Africa to China (Fig. 3d, g). This suggests the African region is also playing the role of a potential exporter, despite dispersal intensity from endemic Africa to the Northern Hemisphere (Florida, Europe, China, and Japan) being only 3.4% of the dispersal intensity seeded from endemic Asia (Fig. 3d, g, j, m).

On top of these two modes, we also identified a mode of spread from fringe areas to endemic areas, such as routes from South China to endemic Asia for all four serotypes (BF ≥ 1000, Fig. 3d, g, j, m), and from Uruguay/North Argentina to endemic America for DENV1 during the pre-pandemic period (BF ≥ 1000, Fig. 3d). Analysis of epidemiological surveillance data from Singapore supports this phylogenetically inferred pathway, with records showing the consistent importation of dengue cases from China (Fig. S15). However, the extent of onward transmission in endemic areas following spread from fringe areas requires more epidemiological and genomic surveillance of outbreak for identifying sequence clusters. Quantitatively, the intensity of the viral movement from South China to endemic Asia was consistently lower and exhibited less seasonality than the primary flow from endemic Asia to South China (Fig. S16; average annual movement intensity: 2.7 vs. 18.4 for all four serotypes, Fig. 3d, g, j, m).

However, our results do not support spread events from Florida or South Europe to other endemic areas (Fig. 3). We found that the proportion of reported dengue cases that were locally acquired in Florida (12.5%) and South Europe (3.1%) from 2010 to 2024 was significantly smaller than that in South China (94.0%; both *P* < 0.001, Chi-square test) (Fig. S17a, d, g). In addition, the overall scale of local dengue incidence in Florida and South Europe was substantially lower than in South China (Fig. S17b, e, h). Consequently, the lack of spread into endemic areas from Florida and South Europe may be attributed to its low domestic incidence. Despite having international travel volumes to endemic regions comparable to South China (Fig. S17c, f, i), the substantially smaller number of locally acquired cases in Florida or South Europe drastically reduces the probability of a viremic individual traveling to and seeding outbreaks in an endemic area.

### Impact of the COVID-19 pandemic on DENV global dispersal patterns

The number of DENV sequences dropped during the pandemic and increased during the post-pandemic period (Fig. S6), whereas sequencing rate remained relatively stable (Fig. S18). Even though genomic surveillance intensity was relatively maintained, we cannot rule out possible biases in genomic surveillance of DENV virus, which warrants the use of multiple sub-sampling approaches. During the COVID-19 pandemic (here defined as the period from 2020 to 2021, after which most countries, except for China, relaxed public health measures), the majority of dispersal routes at the sub-location level were disrupted, especially internal dispersal within each endemic area (Fig. 2b, e, h, k). The dispersal routes seeded from endemic areas (that is, from endemic America to Florida, USA and from endemic Asia to China and Japan) were still identified, albeit with lower dispersal intensity than during the pre-pandemic period (for example, endemic Asia-to-China: 18.4 vs. 1.7). Less extent of support for spread from South China to endemic Asia were also identified for DENV1, DENV2, and DENV3 (Fig. 3e, h, k), robust to sub-sampling scheme (Fig. S12e, h, k). In the post-pandemic period (2022–2025), endemic America appeared to play a more important role in disseminating DENVs into Florida, USA (for all four serotypes) and to South Europe (DENV2 and DENV3) (Fig. 3f, i, l, o).

The COVID-19 pandemic remarkably altered the dynamics of viral net exports from endemic source regions. We observed a substantial reduction in net exports from endemic Asia during the pandemic period (Fig. S19), a trend likely associated with the region’s stringent and prolonged mobility restrictions (Fig. S20). While exports from Asia resumed in the post-pandemic era for most serotypes, they did not for DENV4. In contrast, endemic America exhibited a notable increase in net exports of DENVs following the pandemic (Fig. S19). In addition, the reduction in global travel also affected how long viral lineages circulated locally. The global tip-associated persistence time (time for tips to leave its sampling location walking backwards in time [32]) for DENV strains slightly increased during the pandemic (Fig. S21). This increase is likely associated with the reduced long-distance human travel, which slowed the rate of viral strain replacement.

### Annual source-sink patterns of global DENV circulation

**Figure 4.**
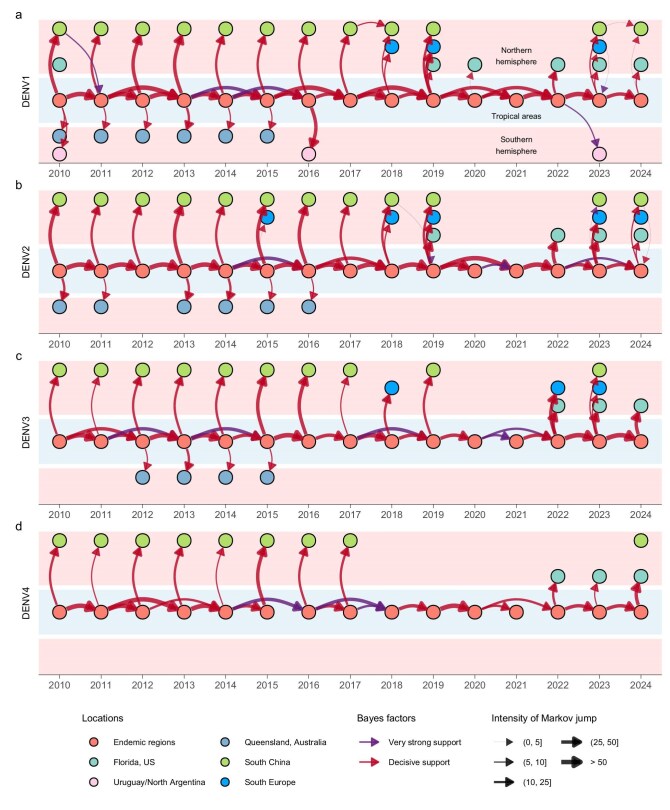
Overwintering migration patterns among geographic locations. (a–d) Estimates of multiple-year migration intensity within and between geographic locations for each serotype, respectively. Some locations in the northern and southern hemisphere lack points due to limited availability of corresponding genetic data. The colored lines represent statistical support for a given viral migration route using BF, where only migration routes with a BF ≥ 100 are shown. The thicknesses of the curves refer to the intensity of the Markov jump event.

The spread from fringe areas to endemic areas can be explained by two potential hypotheses: (i) some of fringe areas are becoming new endemic areas; (ii) fringe areas are still acting as non-endemic areas but with a reverse spread pattern into endemic areas. To investigate this and evaluate a potential global source-sink dynamic, we analyzed an additional genetic dataset incorporating both temporal and spatial information into phylogeographic demes. Our results support a source-sink model, showing continuous viral movement across successive years within endemic regions. These endemic sources, in turn, frequently seed annual outbreaks in the fringe ‘sinks’ on the temperate areas (Fig. 4). Although this is the general pattern, our results also show that DENV1 can occasionally overwinter (persistence of lineages across consecutive seasons) in South China (from 2017 to 2018; from 2023 to 2024, Fig. S22), a sign of endemicity potential; nonetheless, they have not yet established sustained year-after-year persistence (Fig. 4a). Taken together, this suggests that local outbreaks in South China can lead to both occasional overwintering and the re-exportation of the virus to endemic areas with a low intensity, which might result in South China being in an intermediate state between endemic and non-endemic conditions. However, to verify endemic transition, it is necessary to perform routine genomic surveillance for locally circulating DENV strains at the interfaces of population and vectors throughout the year in South China.

The presence of a source-sink model is further supported by the patterns of tip-associated persistence time (Fig. S21). Tip-associated persistence times of all DENV serotypes in endemic Asia and America were all more than 5 years, compared with ∼1 year in other fringe or non-endemic areas (Fig. S21). Longer persistence of viral strains in endemic regions might suggest temporal continuity of viral evolution (evolve locally for a long time period), which thus have the potential to play source roles in maintaining annual epidemics.

### Establishment of annual epidemics in China

**Figure 5.**
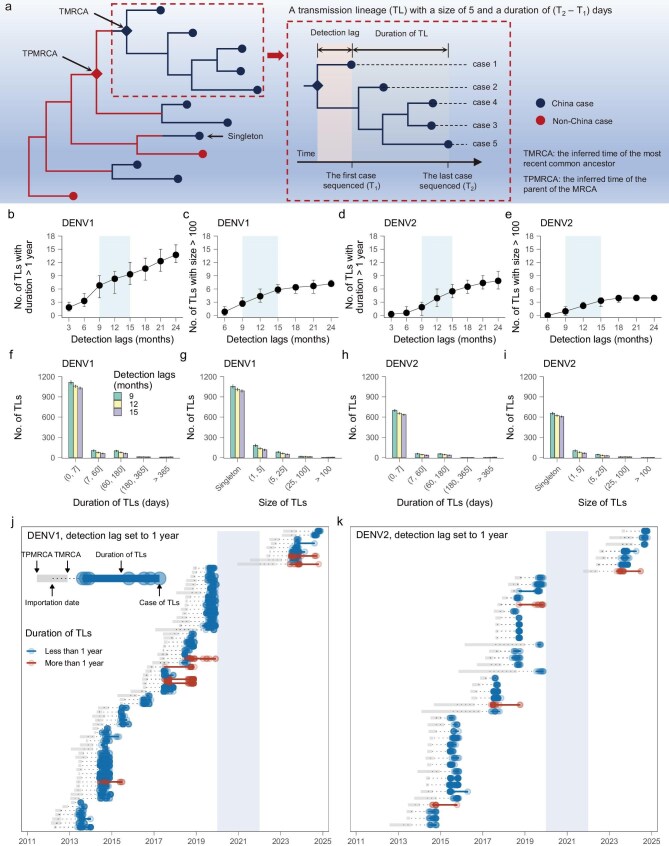
Spatiotemporal distribution of DENV1 and DENV2 transmission lineages (TLs) in China. (a) Schematic diagram illustrating how TLs were identified and how related terms (for example, size, duration) were defined. (b–e) The number of TLs with a duration of >1 year or a size of >100 detected when setting a different detection lag (the time between any ancestor in the deme and the next sample). The light-blue shaded area represents the lag ranges for the following analyses. (f, h) Distribution of duration of DENV1 or DENV2 TLs in China using different detection lags. Error bars represent the 95% HPD intervals of these durations across the posterior tree distribution. (g, i) Distribution of DENV1 or DENV2 TL sizes in China using different detection lags. Error bars represent the 95% HPD intervals of these sizes across the posterior tree distribution. (j) Distribution of all detected DENV1 TLs in China. Gray lines represent the intervals of two inferred key time points [TMRCA (time of the most recent common ancestor) and TPMRCA (time of the parent of the MRCA)], and the importation date of each TL was estimated by taking the midpoint between the internal node corresponding to the introduction (TMRCA) and its parent node (TPMRCA). Only TLs with size >5 and duration >1 week are shown. The light-purple shaded area represents the COVID-19 pandemic period, defined as January 2020 to December 2021. (k) Same as (j) but for DENV2.

Although previous results have shown that local dengue epidemics in fringe areas are mainly seeded from endemic regions and occasionally overwinter, the process of how annual epidemics are established in non-endemic regions still remains unclear. We thus used all DENV1 and DENV2 sequences in China along with curated global background genetic sequences to analyze the spatiotemporal distribution of transmission lineages (TLs, defined as cluster of sequences in the phylogeny that descends from a non-China internal node [33], Fig. 5a) in China.

Based on the previous phylogeographic outputs, the geographic source of viral influx into China was primarily estimated to be endemic Asia (Fig. S23), consistent with 93.1% (1571/1688) of imported cases (78.8% in Central China and 94.3% in South China) had travel history from endemic Asia in China (Table S5). Endemic Africa and America occasionally contributed to an extent of less than 20% importation (except for DENV3 in 2023, Fig. S23). Once the virus was imported into China, the size and duration of transmission lineages varied substantially. The identification of TLs from phylogeny relies on the setting of allowed detection lag where a longer permissible lag would lead to the capture of larger and longer TLs (Fig. 5b–e). Here, we considered a range of 9–15 months as reasonable intervals from case importation to detection for a non-pandemic pathogen. In general, when setting a detection lag of 1 year, 82.1% (1012/1232) and 83.1% (623/750) of DENV1 and DENV2 transmission lineages in China were singletons (Fig. 5g, i), meaning no further sequence clustered together under the current sub-sampling scheme. It should be noted that estimates of TL might be affected by the heterogeneity of genomic surveillance [33,34]. The transmission lineages with duration of less than 1 week accounted for 86.0% (1060/1232) and 87.5% (656/750) for that of DENV1 and DENV2, respectively (Fig. 5f, h). Despite this, there were still a total of 4 and 2 TLs had a size exceeding 100 sequences, and 9 and 4 TLs lasted for more than 1 year, for DENV1 and DENV2, respectively (Fig. 5f–i). These findings align with the possibility of overwintering spread in China (Fig. 4). However, given that the temporal distribution of cases along these long-persisted TLs were sparse (Fig. 5j, k), it merits more genomic investigations whether cases from these same TLs can be continuously detected between consecutive epidemics. In addition, individual transmission lineages showed high temporal heterogeneity, and the onset of the COVID-19 pandemic led to a ∼2-year interruption of onward transmission (Fig. 5j, k).

### Multiple covariates as predictors of DENV spread

**Figure 6.**
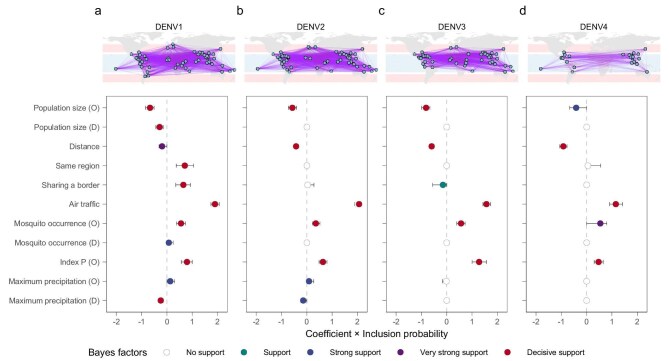
Predictors of DENV spread at the country scale. (a–d) Top panels show the spatial distributions of countries/territories included in the analyses for DENV1 (*n* = 54), DENV 2 (*n* = 54), DENV3 (*n* = 47), and DENV4 (*n* = 24), respectively. Bottom panels show the posterior summaries of the product (reported as log effect size) of the constant through-time predictor inclusion probability and the log predictor coefficient for each serotype. The colored points represent statistical support using BF. Points and ranges represent the posterior mean and 95% HPD intervals, respectively. Location specific predictors were included as both origin (O) and destination (D) predictors of the pairwise transition rates except for index P. Review drawing number: GS 京(2026)1788号.

The spatial dissemination of DENV in Asia and Americas was shown previously to be associated with human mobility [6,25]. To identify more potential drivers of DENV spread at global scale within fine spatiotemporal resolution, we used a multi-epoch (that is, multiple discrete time windows, each corresponding to a 2–3 year interval, see Methods section) Bayesian phylogeographic generalized linear model (GLM) at the country level. In general, the relative importance and direction of predictors appears to be largely consistent across serotypes (Fig. 6). As expected, we found that air travel was a predictor of global DENV dissemination, with positive log effect sizes of 1.89 [95% highest posterior density (95% HPD) = 1.74–2.06] for DENV1, 2.06 (95% HPD = 1.89–2.20) for DENV2, 1.57 (95% HPD = 1.43–1.73) for DENV3, and 1.15 (95% HPD = 0.91–1.42) for DENV4 (Fig. 6). Distance negatively impacted DENV spread, with effect size of −0.19 (95% HPD = −0.31 to 0), −0.42 (95% HPD = −0.50 to −0.34), −0.60 (95% HPD = −0.69 to −0.50), and −0.92 (95% HPD = −1.06 to −0.77) for each serotype, respectively (Fig. 6). However, the negative effect of distance is less pronounced in Asia (Fig. S24), which might be associated with more diverse and rapid human mobility networks there, effectively leading to a smaller friction of absolute distance relative to the Americas. Additionally, mosquito occurrence probability (a composite for *Ae. aegypti* and *Ae. albopictus*) at the origin location were positively associated with DENV spread, with effect sizes of 0.56 (95% HPD = 0.38–0.73), 0.36 (95% HPD = 0.22–0.52), 0.57 (95% HPD = 0.40–0.73), and 0.54 (95% HPD = 0–0.80) for each serotype (Fig. 6). High index P at the origin location were also associated with a strong DENV spread into other locations, which was consistent across serotypes. In contrast to a positive link with mosquito occurrence, we found a negative association between DENV spread and population size at the origin (Fig. 6). Finally, we did not find conclusive evidence that maximum precipitation was associated with DENV spread, although we found associations for DENV1 (0.13, 0–0.31) and DENV2 (0.09, 0–0.27) (Fig. 6). These findings suggest a complex dynamic between local suitability, mosquito occurrence, human population size, and human mobility.

## DISCUSSION

By integrating epidemiological and phylogenetic evidence, our study suggests that global DENV circulation follows a source-sink dynamic, where persistent viral reservoirs in the tropics annually seed epidemics in temperate regions. We also revealed heterogeneity in both within-region and across-region spread among the endemic regions, indicating that viral maintenance is relatively independent within each endemic area. Among those fringe areas, we found that South China was on a path toward endemicity, as shown by a low-intensity viral flow occurring from South China to endemic ‘sources’ and occasional DENV1 overwintering phenomena. Furthermore, we showed that these global dispersal patterns were remarkably reshaped by the COVID-19 pandemic, which disrupted established pathways and altered the relative contribution of different regions to global DENV circulation.

Our conclusion that global DENV circulation operates as a source-sink system is supported by several convergent lines of evidence from our analysis. First, endemic tropical regions consistently function as persistent viral sources, as shown by their capacity for year-to-year viral maintenance and their significantly longer lineage persistence times compared to temperate regions. Second, temperate regions act as epidemic ‘sinks’, with our data showing that local outbreaks in those regions are typically transient, highly seasonal, and reliant on recurrent viral introductions from these source regions. Third, this constant source-sink viral traffic is connected by human mobility and jointly driven by vector and climate, as our GLM analysis identifies air travel, mosquito occurrence, and index P as key predictors of DENV spread. In contrast to endemic regions in Asia and the Americas, endemic Africa appears to play a lower role as a global source population. This might be linked to the distinctive bionomics of *Ae. aegypti* populations in Africa [35]. However, more comprehensive surveillance data would be needed to support this claim.

Previous studies have suggested that silent human transmission and vertical transmission or overwintering in mosquito vectors might result in viral persistence between epidemics [18,36]. Several mechanisms can contribute to enabling tropical regions to act as persistent sources, such as climatic conditions [37], urbanization [38], living habit (for example, rainwater tanks) [39], and quick replenishment of susceptible human hosts due to high birth rates [18]. It is still unclear whether the presence of sylvatic cycles in Africa and Asia is a contributing factor to DENV persistence, although previous studies suggest that sylvatic DENV is maintained there [40] and sylvatic strains may not require significant adaptation to infect humans [41,42]. Enhanced surveillance for sylvatic DENV strains will be crucial to further elucidate the interface between sylvatic and human cycles, and their contributions to DENV persistence and global circulation. Furthermore, the geographical boundaries defining ‘source’ and ‘sink’ regions are not static, as illustrated by the case of South China. Climate and urbanization are likely contributors to the expansion of the spatial range of mosquito vector species suitability [12,43]. Therefore, this source-sink framework must be viewed as an evolving paradigm reflecting dynamic global changes.

The identification of spread from South China to endemic Asia has important implications for understanding the endemicity of fringe areas and global dengue control strategies. The combination of recurring outbreaks and spread into endemic areas raises concerns that fringe regions such as South China are on a path toward endemicity, especially in the context of local conditions for vector viability. Such a transition would expose hundreds of millions of immunologically naive individuals to DENV infection risk and increase the global burden of dengue. In addition, emphasis has been historically placed on viral flow from endemic to fringe areas [44] or within endemic regions [45]. However, once DENV seeds local outbreaks in fringe areas, subsequent population mobility can lead to viremic travelers reintroducing the virus into endemic regions. The dispersal dynamic seeded from South China underscores the importance of monitoring not only endemic foci, but also the fringe of endemic regions, from which we can also constantly capture the dynamics of source-sink boundaries. Recent study revealed that back-to-Africa migration of the invasive subspecies *Ae. aegypti* introduced insecticide resistance and anthropophily into Africa with recent dengue outbreaks [46]. As viruses evolve under distinct ecological pressures (for example, host immunity) [47,48] and mosquitoes face varying selective pressures (for example, resistance from insecticide exposure) [3], it underscores the need for surveillance systems capable of detecting the emergence of novel viral phenotypes in fringe and non-endemic areas and their re-introduction into endemic regions.

Our analysis has shown that local DENV outbreaks in China were predominantly triggered by imported cases, with occasional overwintering transmission that eventually disappeared without *in-situ* evolution [49]. The magnitude and duration of local transmission lineages varied substantially, with large-scale DENV outbreaks only made possible by a combination of interacting factors, including the number of imported cases, mosquito density, and temperature [50]. Although less well-documented, interactions with co-circulating arboviruses (for example, viral interference, virus–virus interactions via antibody effects) [51] might also influence outbreak size or persistence. Investigating the molecular evolutionary characteristics and potential host-adaptive mutations of persistent and overwintering lineages in the future could provide key insights into the biological drivers underpinning these global viral dispersal patterns. While China remains vulnerable to recurrent introductions, current evidence might support that South China is transitioning into an intermediate state between endemic and non-endemic conditions. However, endemic transition must require a mosquito ecosystem that can sustain virus transmission, virus circulation that can persist over time, and most importantly, the persistence of viral strains in human population across multiple years. Although we observed an endemic-like pattern in South China, evaluations of endemicity should be conducted at fine spatial scales to properly capture such heterogeneities, as China spans a range of climatic conditions and vector abundance, like other temperate regions (for example, Europe, the contiguous USA, and Australia).

While coordinated global surveillance and control strategies are essential for reducing DENV burden, the practical definition of operational intervention triggers depends heavily on localized public health infrastructure and resources. The quantitative relationships identified in this study provide the empirical parameters necessary to inform these frameworks. Specifically, our models quantify the conditions under which climate suitability (Index P) and viral importation intensity acts as drivers of viral dispersal. These metrics lay a data-driven foundation for the creation of local-scale prospective early warning systems.

Our study has several limitations. First, sampling biases are inevitable in phylodynamic analyses [52]. To mitigate these biases, curated genetic datasets were subsampled using either infection-informed or spatially even sampling strategy, which were shown to be relatively robust to the issues such as uneven distribution of genomes [53]. Second, heterogeneous genomic surveillance across regions limited the inclusion of underrepresented areas such as southern and northern Africa. Although we included available DENV sequences from Africa, the scarcity of data from 2010 to 2015 may have affected our phylogeographic reconstruction. Third, fine-scale analyses were constrained by the geographical resolution of available virus genome data and model capabilities. We performed our analyses at three different spatial levels as fine-scale as possible and our results are robust to some changes in the spatial scale of analysis (for example, Fig. 3a–c). Models that integrate phylogenetic analyses with detailed mathematical transmission models could help bridge this gap in the future. Fourth, the transmission lineages described here do not directly equate to epidemiological transmission chains or clusters, due to differences in spatial scale and the impact of sampling biases [33]. Detailed epidemiological investigations could help bridge this gap. Fifth, while our study provides an empirical evaluation of global DENV circulation and explores the primary independent drivers of viral dispersal, but a deeper understanding of how climate, urbanization, population immunity, and human mobility (including land and airline mobility) jointly affect these dynamics would require further causal inference and mechanistic approaches to account for multi-factor interactions [3].

## CONCLUSIONS

Improving our understanding of global DENV circulation dynamics has important implications for dengue preparedness and control. Our study provides an empirical evaluation of global DENV circulation over the past 15 years. By integrating multiple data streams from endemic, fringe, and non-endemic regions, we found evidence of a source-sink dynamics, but the roles of individual countries within endemic areas as the source population need further investigation. We found most areas on the fringe of endemic transmission act as ‘sink’, but South China seems to be in an intermediate state between endemic and non-endemic conditions with evidence of spread back to endemic ‘sources’ and occasional overwintering. Such dynamics created a fully interconnected global dispersal system and emphasized an evolving paradigm of source-sink patterns. Understanding this complex dynamic is essential, as monitoring the future expansion and endemic establishment of dengue requires coordinated global surveillance.

## METHODS

Detailed Methods are available in the Supplementary data.

## Supplementary Material

nwag372_Supplemental_File

## Data Availability

The findings of this study are based on sequences available on GISAID and NCBI up to November 29, 2025. Global dengue surveillance data were available from the WHO Global Dengue Dashboard (https://worldhealthorg.shinyapps.io/dengue_global/). Dengue case data in France, Italy, and Spain were collected from the European Centre for Disease Prevention and Control (https://atlas.ecdc.europa.eu/public/index.aspx), which were based on data provided by WHO and Ministries of Health from the affected countries. Dengue surveillance data in Taiwan, China were available from Taiwan Infectious Disease Statistics System (https://nidss.cdc.gov.tw/en/Home/Index?op=4). Dengue surveillance data in Zhejiang, Fujian, Yunnan, and Guangdong, China were provided by the local institutes. Dengue surveillance data in the US (including territories) were provided by ArboNET under a data use agreement with the CDC Division of Vector-Borne Diseases (https://www.cdc.gov/vector-borne-diseases/php/arbonet/index.html). Australian dengue data were collected from the National Notifiable Disease Surveillance System (https://nindss.health.gov.au/pbi-dashboard/). Dengue case data in Uruguay were collected from Ministry of Public Health, Uruguay (https://www.gub.uy/ministerio-salud-publica). The origin-destination passenger air traffic data were provided by Official Airline Guide (OAG) Ltd. (https://www.oag.com) through a data sharing agreement. All codes and xml files used for phylogenetic and phylogeographic analyses are available here: https://github.com/zychenfd/global_dengue_project.
