# Supplementary material for: Global source-sink dynamics of dengue viruses and epidemic establishment in areas on the fringe of endemic transmission
Source: Natl Sci Rev. 2026 Jun 15;13(14):nwag372. doi: 10.1093/nsr/nwag372 (PMC13389948; doi:10.1093/nsr/nwag372)
Supplement: nwag372_Supplemental_File [file nwag372_supplemental_file.docx]

Supplementary Information for

**Global source-sink dynamics of dengue viruses and** **epidemic establishment in areas** **on the fringe of endemic transmission**

Zhiyuan Chen^1^, Marta Giovanetti^2,3^, Zachary J. Madewell^4^, Andi Sun^1^, Wenzhi Li^1^, Chunmin Li^5^, Haoyin Yu^5^, Jimin Sun^6^, Liyun Jiang^7^, Pengzhe Qin^7^, Xinwei Wu^7^, Marco Ajelli^8^, Hongjie Yu^1,9,10^*

**Affiliations:**

1. School of Public Health, Key Laboratory of Public Health Safety, Ministry of Education, Fudan University, Shanghai, China
2. Department of Science and Bio-Technology, Università Campus Bio-Medico di Roma, Rome, Italy
3. Oswaldo Cruz Institute, Oswaldo Cruz Foundation, Rio de Janeiro, Brazil
4. Division of Vector-Borne Diseases, Centers for Disease Control and Prevention, San Juan, Puerto Rico
5. Yunnan Institute of Parasitic Diseases, Yunnan Provincial Key Laboratory of Vector-borne Disease Control and Research, Yunnan International Joint Laboratory of Tropical Infectious Diseases, Kunming, Yunnan, China
6. Zhejiang Provincial Center for Disease Control and Prevention, Hangzhou, Zhejiang, China
7. Guangzhou Center for Disease Control and Prevention (Guangzhou Health Supervision institute), Guangzhou, Guangdong, China
8. Laboratory for Computational Epidemiology and Public Health, Department of Epidemiology and Biostatistics, Indiana University School of Public Health-Bloomington, Bloomington, IN, USA
9. Shanghai Institute of Infectious Disease and Biosecurity, Fudan University, Shanghai, China
10. Department of Infectious Diseases, Huashan Hospital, Fudan University, Shanghai, China

*Corresponding author: yhj@fudan.edu.cn

# Methods

## Spatiotemporal scale

Given the boundaries of endemicity defined by the WHO and United States CDC ^1,2^, we divided the world into endemic areas and non-endemic areas (**Table S1**). For areas adjacent to endemic areas, we established a quantitative metric to define “fringe areas”. Briefly, a fringe area must simultaneously meet the following two criteria: i) an endemic criterion, where the proportion of local cases exceeds 5% and at least 50 local DENV cases have been reported in at least one year over the most recent 15-year period; and ii) a non-endemic criterion, where the median annual duration of local transmission is less than 7 months. To estimate the duration of local transmission, we calculated the proportion of monthly DENV cases relative to the annual total cases per year, defining active transmission as any month where local cases exceeded 1% of the annual total. The median annual duration across epidemic years was used as a proxy for local transmission duration (**Fig. S1, Table S3**). Because monthly local case data were unavailable for Queensland, Australia, we substituted the monthly case metric with the index P (using a threshold of 0.5) to represent the intensity of local transmission, given its correlation with empirical transmission patterns observed in other locations (**Fig. 1b-h)**. Accordingly, five “fringe areas” were defined, including the United States (Florida), South Europe (Italy, France, Spain), South China (Guangdong, Yunnan, Zhejiang, Guangxi, Hainan, Fujian, Taiwan), Australia (Queensland), and Uruguay/North Argentina (**Fig. 1a**). Other regions adjacent to endemic areas (e.g., the southern and northern part of Africa) were excluded due to limited availability of genetic sequences.

The endemic areas were further subdivided into endemic America, endemic Africa, and endemic Asia. Given an unclear endemicity division in Africa partly due to the surveillance systems, we defined endemic areas in Africa based on historical endemicity classification for those areas with genetic data publicly available (**Table S2**). Three non-endemic areas included West Europe (Germany, Switzerland, Belgium), Central China, and Japan. Territories were classified based on geographical rather than administrative boundaries (e.g., Puerto Rico was classified under endemic America despite being a US territory). For all analyses, the temporal scale was set from January 2010 to June 2025. This range was chosen to ensure a relatively stable molecular signal while also accounting for the limited availability of sequences prior to 2010.

## Data sources

### *Epidemiological surveillance*

Monthly-resolution dengue cases disaggregated by travel status (locally acquired or travel-associated) were collected through multiple data sources. Province-level surveillance data from 2010 to 2025 in South China, distinguished by travel status for Guangdong, Yunnan, Zhejiang, Fujian, and Taiwan were obtained either via local CDC or direct data sharing. US data (including territories) were provided by ArboNET (<https://www.cdc.gov/mosquitoes/php/arbonet/index.html>), the national arboviral surveillance system. Australian dengue data were collected from the National Notifiable Disease Surveillance System (<https://nindss.health.gov.au/pbi-dashboard/>), while yearly travel-status data were retrieved from Sohail et al ^3^. Dengue case data in South Europe were collected from the European Centre for Disease Prevention and Control (<https://atlas.ecdc.europa.eu/public/index.aspx>). Dengue case data in Uruguay were collected from the Ministry of Public Health, Uruguay (https://www.gub.uy/ministerio-salud-publica). Surveillance data in other locations were primarily sourced from the WHO Global Dengue Dashboard as of December 9, 2025 ^4^, which consolidates and reports local case data; Réunion Island was excluded due to a lack of travel-status breakdown data. All time-series data were aligned to monthly resolution according to WHO processing criteria ^4^.

### *Genetic data*

All available dengue virus genomes were downloaded from the Global Initiative on Sharing All Influenza Data (GISAID) ^5^ and the National Center for Biotechnology Information (NCBI) ^6^ as of November 29, 2025 and processed through the following pipelines. First, genomes with sequence length shorter than 1,000 nt were removed. To verify virus typing, we used Nextclade to assign serotypes ^7^, then re-assigned serotype and genotypes using a serotype-specific pipeline based on the latest dengue virus lineage nomenclature system ^8^. Genomes that were unassigned or inconsistently typed were discarded. For each serotype, genome sequences were aligned using MAFFT v7.505 ^9^ based on reference sequences (NC_001477.1, NC_001474.2, NC_001475.2 and NC_002640.1) for DENV1, DENV2, DENV3 and DENV4, respectively.

Since the majority of genomes were incomplete, we selected the structural gene region (encoding the capsid, membrane, and envelope proteins) for downstream phylodynamic analyses. This region provides longer sequences than the envelope gene alone, offering more stable molecular signals. We excluded sequences with less than 60% coverage or more than 5% ambiguous sites in the structural gene. To maintain consistency, only sequences from urban-cycle hosts (*Homo sapiens*, *Ae. aegypti*, and *Ae. albopictus*) were retained, excluding sylvatic (e.g., primate-derived) or host-unknown sequences. We also removed strains flagged as excluded isolates (e.g., overly divergent, duplicates or outliers) per the Nextstrain dengue configuration ^10^. We corrected errors in the entry of sampling dates and locations based on genome-related literatures where available. We discarded sequences lacking metadata on collection date or sampling locations, as well as those where the entry of date and the date in the virus name did not match. Sampling locations were then manually reviewed and categorized according to our spatial scale. Finally, we identified and removed the duplicated sequences in each dataset (GISAID or NCBI) if they had the same isolate names, and further performed de-duplication between datasets with the following criteria: i) sequences have the same isolate names or; ii) sequences have the same sampling location (level-0 boundary), collection date, and whole consensus genome sequence, similar to Chen et al ^11^. Priority was given to metadata in NCBI when conflicting data existed. Additionally, we manually checked other potential duplicates that were not captured by the above procedures due to slight changes in isolate names of the same strain when repeatedly depositing them within and between datasets. An overall flowchart of the collection and removal of genetic data is presented in **Fig. S4**.

### *Travel history of genetic data*

Inclusion of recent travel history of cases into phylogeographic inference has been proven to improve the accuracy of estimates for travel-associated infectious diseases ^12,13^. Therefore, we systematically collected and collated travel history information for the sequences retrieved from DENV cases through the following methods. First, we retrieved and double-checked all available metadata information of genomes from GISAID and NCBI. We refined the sampling locations and associated travel history when either was missing or inaccurately recorded, especially for those whose sampling locations were only recorded as travel history or travel history were not correctly linked. Second, we conducted a literature review and extracted detailed travel information from 68 genome-related publications (55 for China, 7 for Europe, 3 for US, 2 for Uruguay/North Argentina, 1 for Australia). For controversial records, priority was given to the information retrieved from the published literature. Third, we directly contacted the authors of submitted genomes to retrieve about the travel-history information, which allowed us to classify an additional 980 genomes (including those genomes labelled as local cases). As a result, travel history data is available for 5,213 out of 8,334 sequences from fringe and non-endemic areas, and the completeness and details of travel history information are presented in **Tables S4-5**.

### *Covariates*

We collected the following covariates to explore their associations with dengue spatial dispersal at the country level given their potential importance in DENV spread and vector competence. Monthly air passenger volume at airport-airport resolution from 2011 to 2024 were provided by Official Airline Guide (OAG) Ltd. through a data sharing agreement. We also downloaded datasets of index P, a proxy representing the climate-driven (temperature and relative humidity) transmission potential of dengue virus transmitted by a single female mosquito ^14^. Precipitation data were derived from the European Centre for Medium-Range Weather Forecasts (ECMWF) Reanalysis v5 (ERA5) ^15^. Mosquito occurrence probabilities of *Ae. aegypti* and *Ae. albopictus* at a spatial resolution of 5 km × 5 km were obtained from a previously established global map ^16^, from which we calculated the total probabilities of *Ae. aegypti* and *Ae. albopictus* occurrences within each location. Population data were downloaded from the United Nations (<https://population.un.org/wpp/>).

## Epidemiologically descriptive analyses

Based on the dengue case datasets, we evaluated the temporal patterns (referred to as seasonality hereafter) of the dengue epidemic by travel status across each spatial scale as defined above. In South China, we included five provinces (Guangdong, Yunnan, Zhejiang, Fujian, and Taiwan) for this analysis due to data availability. To account for heterogeneity in surveillance intensity across locations and years, we first calculated the proportion of monthly dengue cases relative to the total annual dengue cases, to indicate the relative epidemic seasonality within a single year. Years with missing data, large negative case values (values greater than -10 are converted to 0; negative values are caused by retrospective adjustments ^4^) in any months, or with zero cases in more than nine months were excluded. In each location, years with fewer than 15 total reported dengue cases were also discarded, as they might lack enough data to reveal detectable seasonal patterns. Data from 2020 to 2022 were removed from this analysis due to COVID-19 pandemic. Next, we grouped the relatively standardized dengue seasonality time series by month and applied a generalized additive model (GAM) to derive an average pattern. A cyclic cubic regression spline with *k* = 12 knots was used as the smoothing function for the proportion, with analysis performed in R version 4.3.3. The seasonal pattern of index P was also estimated in a similar manner. In addition to displaying an average pattern, we also presented all original patterns of case and index P for each country or state in each year (**Fig. S3**).

## Initial phylogenetic collations

We performed a step-by-step procedure to remove divergent genetic sequences that could interfere with downstream phylogenetic and phylogeographic reconstruction. First, we constructed an approximate maximum likelihood (ML) tree for each dengue serotype in FastTree 2.1.11 ^17^, from which long branches were identified and removed. Second, we adopted the RDP5 ^18^ to detect and remove potential recombinants, where only those identified by at least five out of seven recombination detection methods were excluded ^19-25^. Third, we generated an ML tree for each serotype using IQ-TREE 2.4.0 ^26^ and used TempEst ^27^ to identify and remove temporal outliers. Fourth, identical genome sequences (post-alignment) collected from the same country were removed keeping only the sequence with the earliest collection, except for the analysis focusing on epidemic establishment in China, where all China-sampled sequences were retained.

Given some sequences with incomplete collection dates (e.g., year-month or year only), we applied a Bayesian estimation approach to resolve their sampling times. These sequences were grouped into several subsets, and combined with a date-complete global background dataset, sampled at up to a certain number of sequences per location per year for each serotype. Samples with incomplete dates had dates of sampling estimated assuming a uniform prior within the known temporal bounds in BEAST X v10.5.0 ^28^, with the detailed settings described below.

## Global phylogenetic and phylogeographic analyses

Here we aim to reconstruct the global dispersal patterns of dengue viruses from 2010 to 2025. To test the robustness of our analyses to spatial resolution and sub-sampling bias, we performed phylogenetic and phylogeographic inference using two sub-sampling schemes across three spatial resolutions. The finest spatial resolution is at sub-location level, most of which correspond to countries/territories but with some exceptions (**Fig. S2**). First, certain neighboring countries/territories were merged due to the low availability of genetic data; for example, we grouped African countries into East Africa, Central Africa, West-Central Africa, and West Africa (**Table S1**). Second, South China was divided into six provinces due to their internal epidemiological variability (**Fig. S2; Fig. 1a**). The second spatial resolution is at sub-regional level in which we further aggregated countries/territories within endemic areas into sub-regional demes and regarded South China as an individual deme (**Fig. S2**). The coarsest spatial resolution is at the regional level, where we defined endemic areas into three demes (endemic America, endemic Africa, and endemic Asia). Notably, the number of demes at a given resolution varies across serotypes due to variations in genome availability.

Two sub-sampling approaches were employed with the sub-sampling unit setting at the sub-location level, and only sub-locations with more than 10 sequences will be used for subsequent analysis. The first is an infection-informed sub-sampling regime where sequences are sampled proportional to the DENV infections in each geographic location. Briefly, we first calculated the serotype-specific infections based on the DENV infections (apparent and inapparent) inferred from cartographic models ^29^ and the proportion of infections attributed to each serotype. The latter was estimated from the proportion of sequenced genomes belonging to each serotype in each country from January 2010 to June 2025, estimated using an uninformative Dirichlet prior in a Bayesian framework. Subsequently, for each serotype, we then set the total numbers of sequence sub-sampled in each geographic location to be proportional to serotype-specific infections (with a minimum number of 80 and a maximum of 200 sequences per location where available). Sequences were randomly and uniformly sampled through time for each sub-location according to these sampling targets. To account for the fact that sequences in more recent years were over-represented, at least five sequences were sampled for each year, where available. During the sub-sampling process, sequences with more complete collection dates were given more sampling weights. We ensured that the total subsampled number of genetic sequences from 2010 to 2025 is less than 3,500 in view of the computational efficiency.

The above sampling regime attempts to align sampling with inferred DENV infections, but this approach may not be appropriate if recent patterns of DENV infections did not follow the past average patterns. Therefore, we adopted a complementary scheme to sub-sample sequences equally across spaces (spatially even sub-sampling). By setting a maximum number (100) of sequences for each deme, inference of transmission process should be driven by the underlying sequence diversity itself, which was also validated as effective to improve the inference in a prior methodological study ^30^. In addition, we further used a down-sampling strategy (a maximum sample size of 50 per sub-location) as a sensitivity analysis (**Fig. S13**).

We then performed the phylogenetic analyses for each sub-sampled serotype-specific genetic dataset. We estimated a ML tree in IQ-TREE 2.4.0 ^26^, under which we inferred the time-calibrated tree in TreeTime v.0.11.4 ^31^. Time-calibrated trees would serve as the starting tree for Bayesian phylogenetic inference to accelerate convergence. We adopted the BEAST X v10.5.0 ^28^ to infer the phylogenetic trees of DENV1, DENV2, DENV3, and DENV4, respectively; BEAGLE library v4 was also used to accelerate its core calculations ^32^. We chose a generalized time reversible (GTR) substitution model with gamma-distributed among-site rate heterogeneity, a Hamiltonian Monte Carlo (HMC) relaxed clock model ^33^, and a Bayesian SkyGrid coalescent prior with a HMC operator ^34^ (with grid points equidistantly spaced in 1-year interval until 2010, and equidistantly spaced in 10-year interval before 2010, similar to Taylor-Salmon et al ^35^). For each serotype, Markov chain Monte Carlo (MCMC) was run for 2-3 independent chains, with 60-200 million iterations sampled every 20,000 steps for each chain. Steps were combined across chains, with the first 10-30% discarded as burn-in. Convergence was assessed in Tracer v.1.7.1 ^36^. We resampled states to yield a total of ∼1000 empirical trees for each serotype.

Using the above posterior trees as empirical tree distributions, we employed an asymmetric, travel-aware, discrete phylogeographic model, assigning sequences to three levels of geographic locations, respectively. Travel histories were incorporated to account for frequent travel-associated transmission events, with methods described in Lemey et al ^12^. Nodes with travel history were placed 1 week before tips with known travel history. A time-inhomogeneous epoch model was also used to rationalize the variations in viral transition rates due to pandemic-related human behavior changes, where we defined three epochs using two cutting points (1 January 2020 and 1 January 2022, as most countries implemented minimal public health measures except for China and a dengue epidemic began to resurge afterwards). We used a Bayesian stochastic search variable selection (BSSVS) method to calculate the Bayes factor (BF) to identify the well supported migration routes, where ≥1,000 was deemed as decisive support, 100 ≤ BF < 1000 as very strong support, 10 ≤ BF < 100 as strong support and 3 ≤ BF < 10 as supported ^37^. In addition, we fixed the epoch-specific indicators associated with the geographic rate of demes that are not represented (owing to zero sequence available in this deme in certain epochs) in indices to zero. Due to the computational cost of inferring a huge number of demes at the sub-location level, we adopted a GLM-diffusion phylogeographic model with only air traffic data as the predictor of relative transition rates to inform inference. This choice is supported by our following results where global spatial dissemination of DENV virus was shown to be associated with human mobility (**Fig. 6**).

From the location-annotated posterior trees per sub-sampling scheme and per spatial resolution, we first extracted posterior summaries of Markov jump (inferred lineage movements between locations) events among locations using TreeMarkovJumpHistoryAnalyzer tool ^38^. Upon this, the average annual net exports (export events minus import events) per location per time period were also estimated. We then estimated the tip-associated persistence time (time for tips to leave its sampling location walking backwards in time) per location per time period used PACT v.0.9.5 (<https://github.com/trvrb/PACT>). Notably, we cannot rule out the possibility of estimation uncertainty of persistence time due to the low number of sampled tips; thus we limited our results to those estimates derived from more than 10 available sequences.

## Estimates of viral migration among spatiotemporal demes

In this section, we aim to explore the potential paths underlying the annual maintenance of dengue epidemics across tropical endemic regions and temperate fringe areas (Florida, South China, South Europe, Queensland, Uruguay/North Argentina). To achieve this, similar to a previous study ^39^, we assumed each geographic location and year as a discrete spatiotemporal deme (e.g., sequences collected in 2010 in endemic region was assigned to “endemic region 2010”), which allows us to incorporate seasonality into the phylogeographic analysis. To account for the well-documented dengue seasonality in temperate regions, we only keep viral isolates sampled from their corresponding epidemic seasons, in which January-July for Queensland (Australia) and Uruguay/North Argentina, as well as June-December for Florida, South China, and South Europe.

We applied an even sub-sampling strategy, selecting up to 30 sequences per spatio-temporal deme from 2010 to 2024, where available. We regarded endemic regions as a whole that encompass endemic America, endemic Africa, and endemic Asia, except for DENV4 where sequences in endemic Africa are rarely available. To stabilize the root of subsequent phylogeographic analysis, we also used the even sub-sampling strategy to randomly select sequences in endemic regions from 2000 to 2009 as another discrete character “endemic region 2000-2009”. Discrete demes with few sequences available (less than 5) were discarded. Given the availability of sequences across serotypes, this scheme finally resulted in a range from 28 (DENV4) to 46 (DENV1) discrete demes. Bayesian phylogenetic trees were inferred under settings similar to the previous section. Subsequently, an asymmetric, travel-aware, discrete phylogeographic model was also used for empirical trees. Due to the addition of a temporal element into deme, epoch model was not applied. In subsequent analyses, we excluded transition events in backwards direction (e.g., from South China 2011 to South China 2010) or spanning more than 2 years (e.g., from Florida 2010 to Florida 2013) as it’s impractical to occur. Furthermore, we retained only those posterior trees in which the root was inferred to "endemic region 2000-2009". To assess the impact of configuring such model structure in phylogeographic inference, we developed an alternative phylogeographic model where the indicators associated with backward-direction transition rates were fixed to zero and the root was fixed to "endemic region 2000-2009". The results remain robust (**Fig. S25**); however, adequate mixing was not achieved for the DENV1 analysis due to the large number of spatiotemporal demes.

Inferred Markov jump events among discrete characters were summarized. For the highly supported (BF ≥ 100) overwintering spread from one year to next year in South China, we further extracted the overwintering transmission event along the maximum clade credibility (MCC) phylogeny (**Fig. S22**), which was summarized with keeping target heights in TreeAnnotator.

## Transmission lineages in China

To explore the annual dengue establishment in non-endemic regions, we reconstructed the annual epidemic dynamics of DENV1 and DENV2 in China through extracting China transmission lineages (TLs), a term proposed by du Plessis et al ^40^. All dengue isolates sampled in South China and Central China from 2010 to 2024 were retained to preserve dense sampling, except for sequences whose sampling location could not be specified down to the province level. Given that the majority of imported dengue cases in China had travel histories to endemic areas (especially endemic Asia) ^41^, all sequences from endemic areas were selected as the global background sequences. Likewise, we randomly selected sequences from endemic regions between 2000 to 2009 to stabilize the root of subsequent phylogeographic analysis.

A standard phylogenetic approach became impractical due to the large number of genetic sequences selected (more than 10,000 sequences). Therefore, we adopted an alignment-free likelihood estimation method implemented in BEAST v1.10.4 to estimate the phylogenetic trees, following a similar approach used for SARS-CoV-2 ^40^. In this approach, the time-calibrated ML phylogeny was used as a starting tree, while the polytomic divergence tree served as a data tree. The tree topology was fixed, but the operators concerning node heights and polytomy resolutions were sampled. Using a Bayesian SkyGrid coalescent prior, with grid points set similarly as above, we ran two MCMC chains of 700-800 million states with sampling every 10,000 states.

An asymmetric, travel-aware, two-state discrete trait analysis (DTA) phylogeographic model was applied to empirical trees, with sequences labelled as “China” and “non-China” respectively. We fixed the phylogenetic root as “non-China” in the model because China is rarely likely to act as the root. Three epochs were used, similar to the previous section. To analyze the establishment and maintenance following introduction events, we used Fertree (<https://github.com/jtmccr1/fertree>) to extract TLs (defined as cluster of China sequences in the phylogeny that descends from a non-China internal node, with details in du Plessis et al ^40^) in China from both the MCC tree and posterior trees separately. The identification of TL was related with the setting of maximum detection lag allowed (the time between any ancestor in the deme and the next sample, **Fig. 5a**). Any introduction with lag greater than this limit would be split into introductions that respect the lag are found. Therefore, we performed a series of sensitivity analyses by changing the allowed maximum detection lag from 3 months to 24 months. The size of TL and the duration of TL detection (i.e., from earliest to latest sampling) were summarized by serotypes.

## GLM phylogeographic inference at the country level

Here we aim to identify predictors that might drive the spread of dengue viruses at the country or territory level (referred to as “country” hereafter). We used a spatially even sub-sampling strategy to randomly select the sequence from 2010 to 2024 for each country (selected up to 100 sequences per country where available). Discrete country demes with fewer than five available sequences were discarded, resulting in 54 countries for DENV1, 54 for DENV2, 47 for DENV3, and 24 for DENV4. Bayesian phylogenetic trees were inferred following the previous standard approach. A travel-aware, discrete, time-inhomogeneous phylogeographic model was applied to the empirical trees, with transition rates between countries modeled as a function of various potential predictors using a GLM. To incorporate time-varying predictors, we adopted the epoch model, dividing the time frame into the following intervals: 2010-2011, 2012-2013, 2014-2015, 2016-2017, 2018-2019, 2020-2021, and 2022-2024. A total of eight predictors were used, either in matrix format (e.g., geographic distance, whether countries belonged to the same region, whether they shared an international border, and air traffic) or as attributes of origin and destination locations (e.g., population size, mosquito occurrences, index P only for origin location, and maximum monthly precipitation at each time interval) were correlated with pairwise transition rates. Given a lag between index P and local cases, we shifted the time series of index P forward by 2 months to reflect the intensity of local cases. A constant through-time predictor inclusion probability and coefficient was applied, and the product of these values was reported as the log effect size, which provides a measure of the predictor’s association with the dengue virus spread. We also used DENV2 as an example to compare the primary drivers across Asia and the Americas.

# Supplementary figures


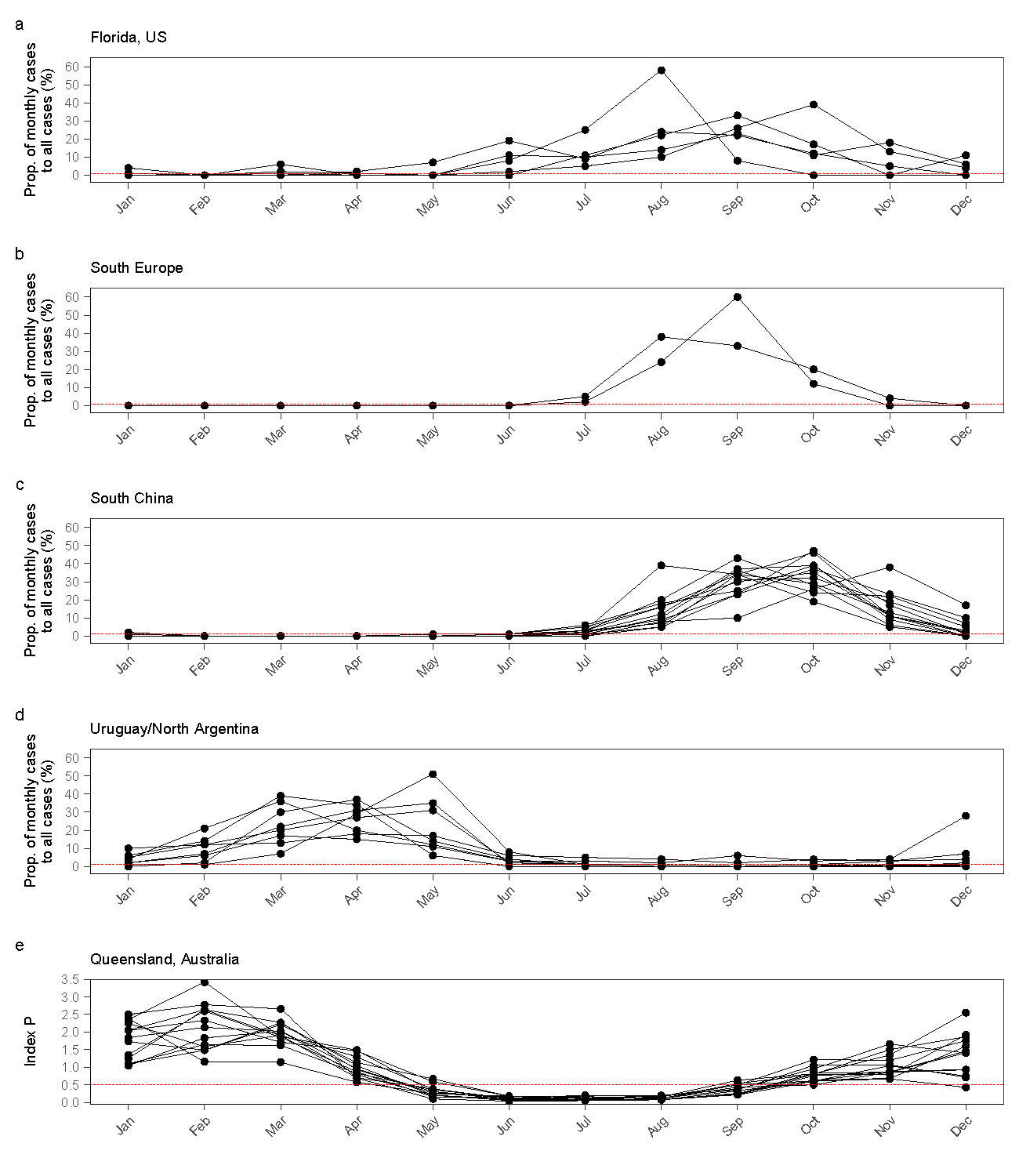


**Figure S1. Annual duration of local DENV transmission in each area.** (**a-d**) Proportion of monthly local cases to all local cases per year in each area. Each line represents a temporal pattern in a specific year. A month was classified as having active local transmission if it accounted for greater than 1% of the annual local cases (dashed red lines); (**e**) Temporal patterns of index P per year in Queensland, Australia. An index P of 0.5 is used as a cut-off to indicate favorable climatic conditions for DENV local transmission (dashed red line).

**
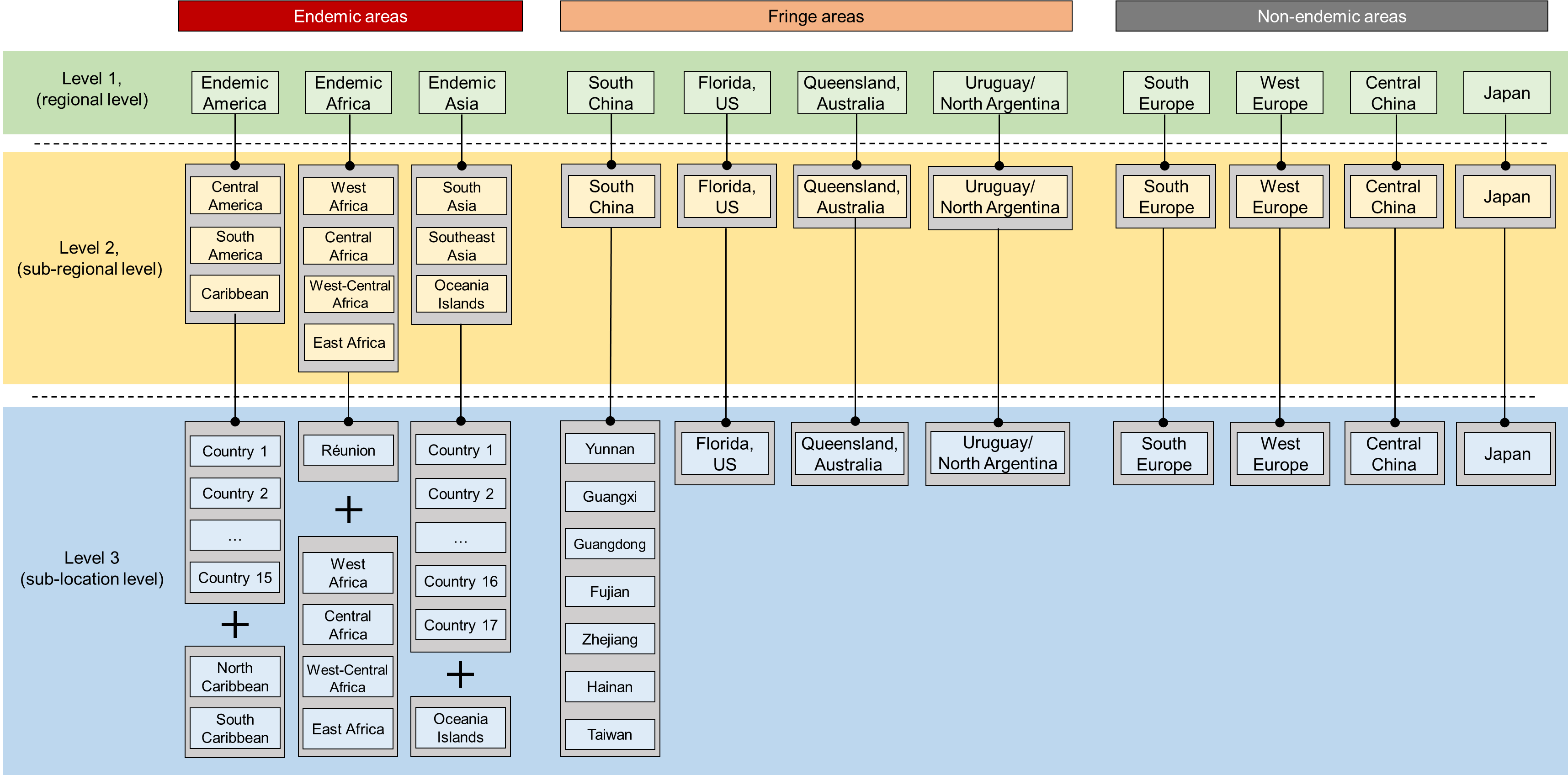
**

**Figure S2. Three spatial scales of global phylogeographic reconstruction: sub-location level, sub-region level, and regional level.** The sub-sampling procedures were performed at the sub-location level, most of which correspond to countries/territories but with some exceptions. For example, some neighboring countries/territories were merged due to the low availability of genetic data there; mainland China was divided into South China (South China was further divided into six provinces) and Central China.

**
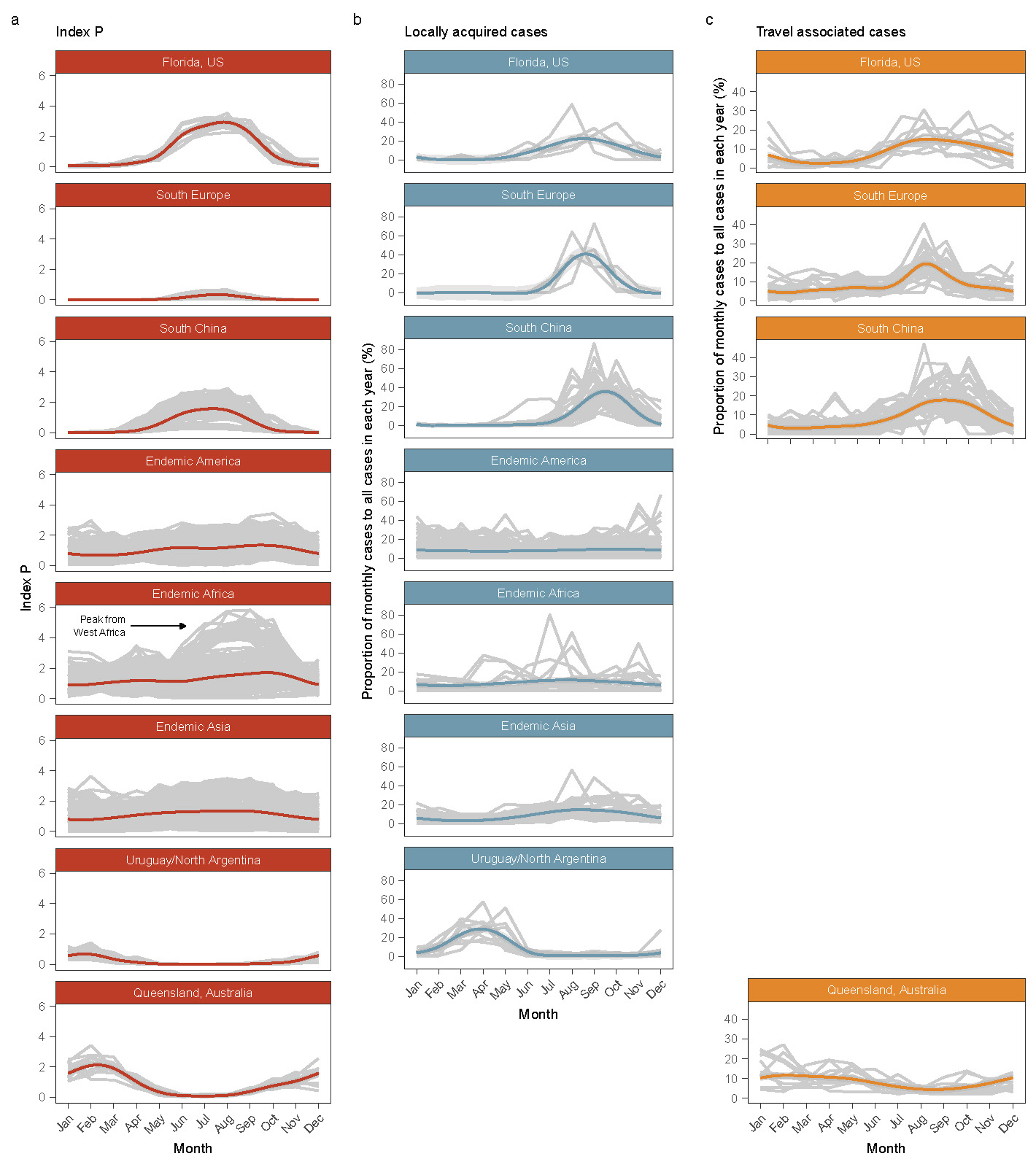
**

**Figure S3. Seasonality of index P and dengue epidemics by travel status across defined geographic locations.** Colored lines represent the average pattern of seasonality fitted by a generalized additive model, while grey lines show the index P/case seasonality for each country or state in each year. The case data for northern Argentina uses data from all of Argentina because monthly case data cannot distinguish between the north and south, and the cases from southern Argentina annually represent a very small percentage. The peak of index P in endemic Africa was observed around Jul-Sep, which was attributed to the pattern from countries in West Africa.

**
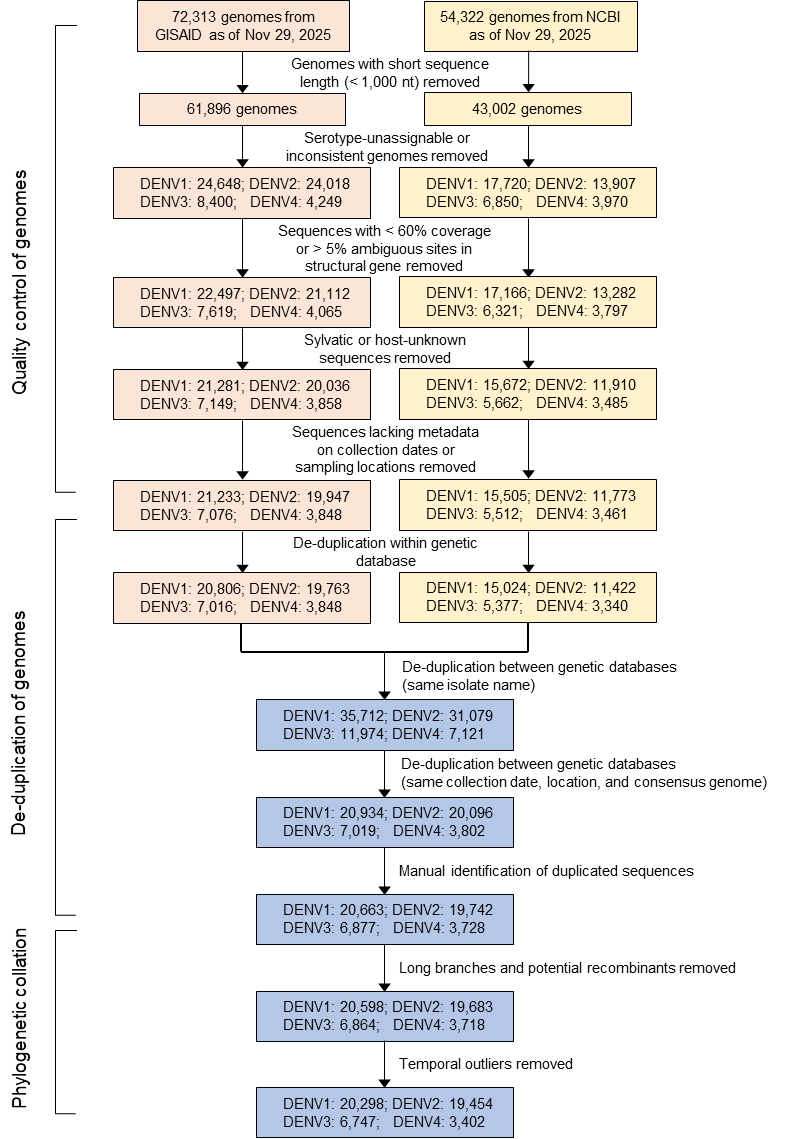
**

**Figure S4**. **Flowchart illustrating how DENV genetic sequences were collated from data available on GISAID and NCBI.** Data were downloaded on November 29, 2025.


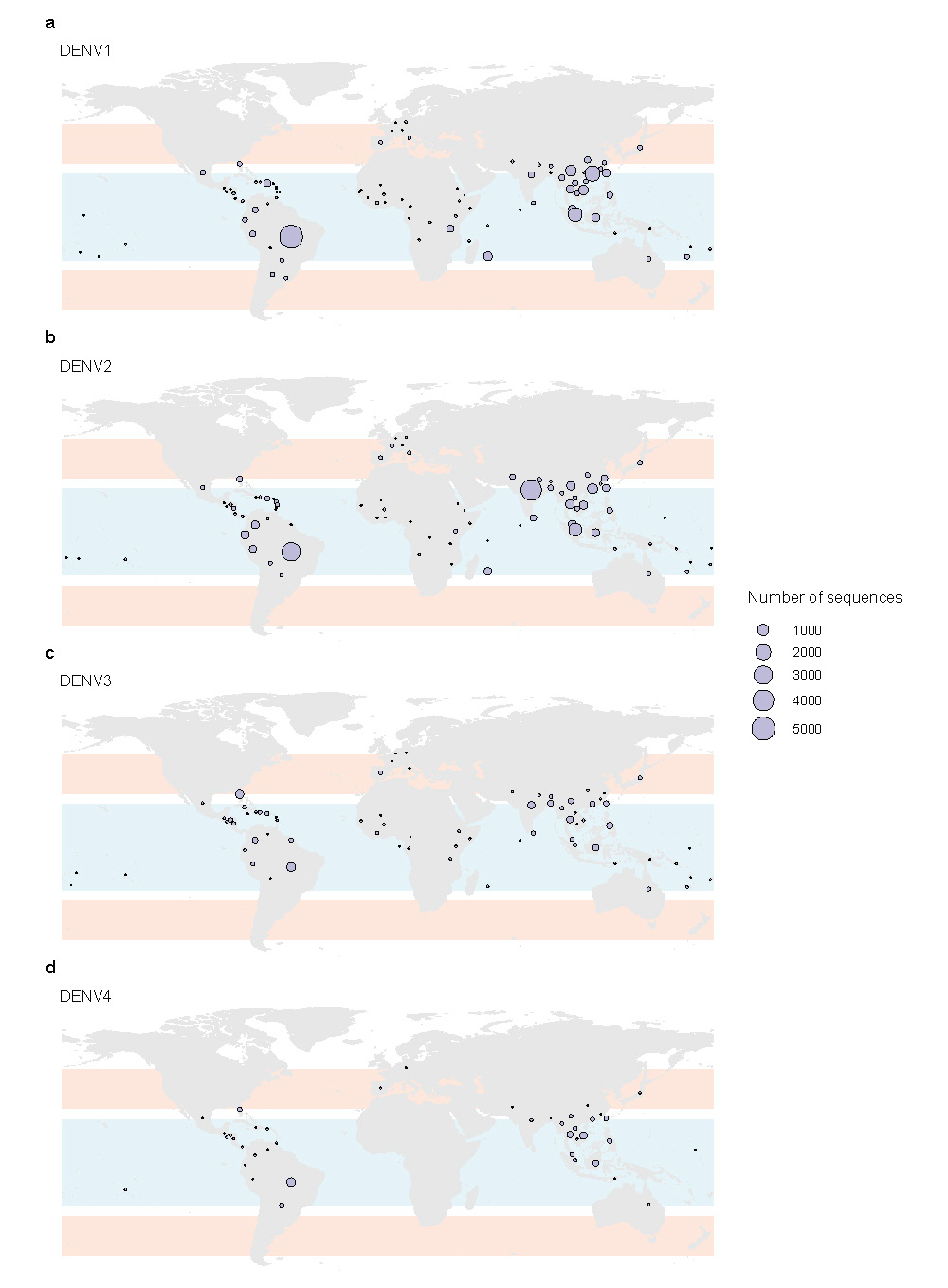


**Figure S5**. **The spatial distribution of DENV genetic sequences collected from January 2010 June 2025 for each serotype.**

**
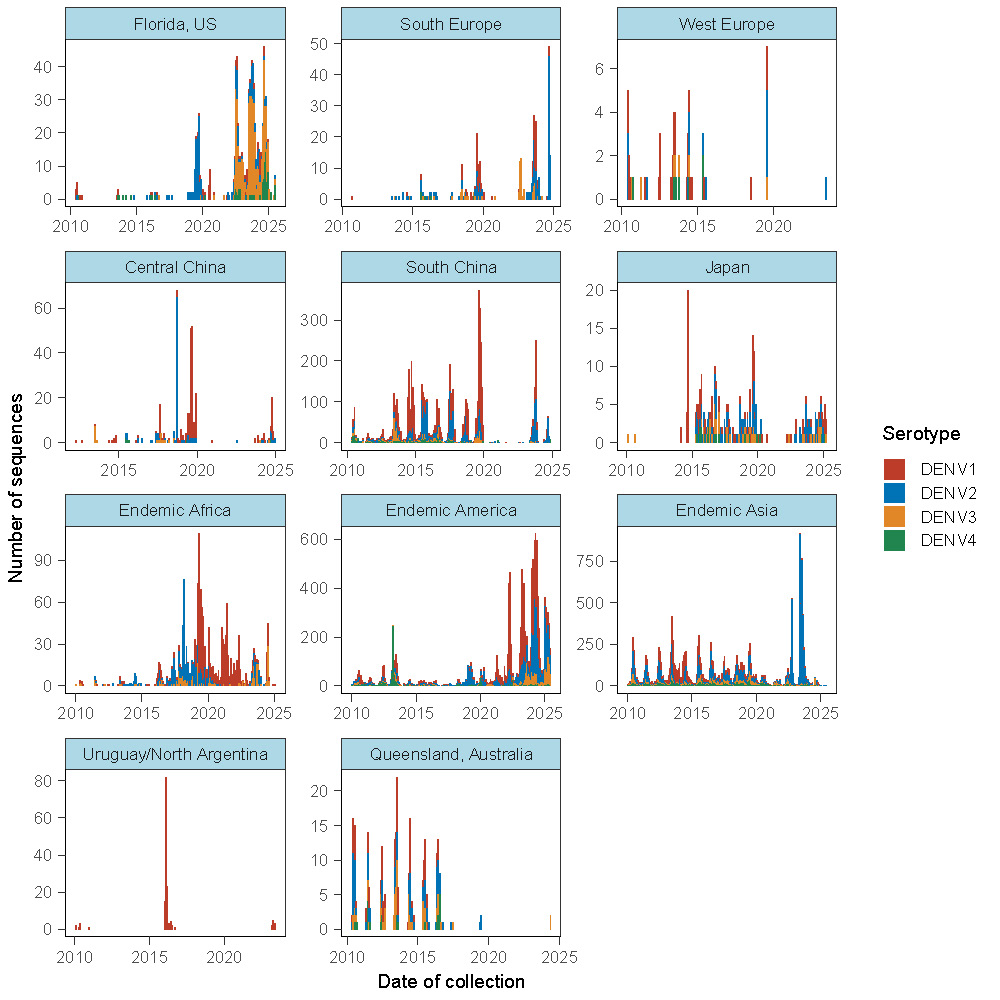
**

**Figure S6**. **The** **temporal distribution of DENV genetic sequences collected from January 2010 June 2025 for each geographic region.**

**
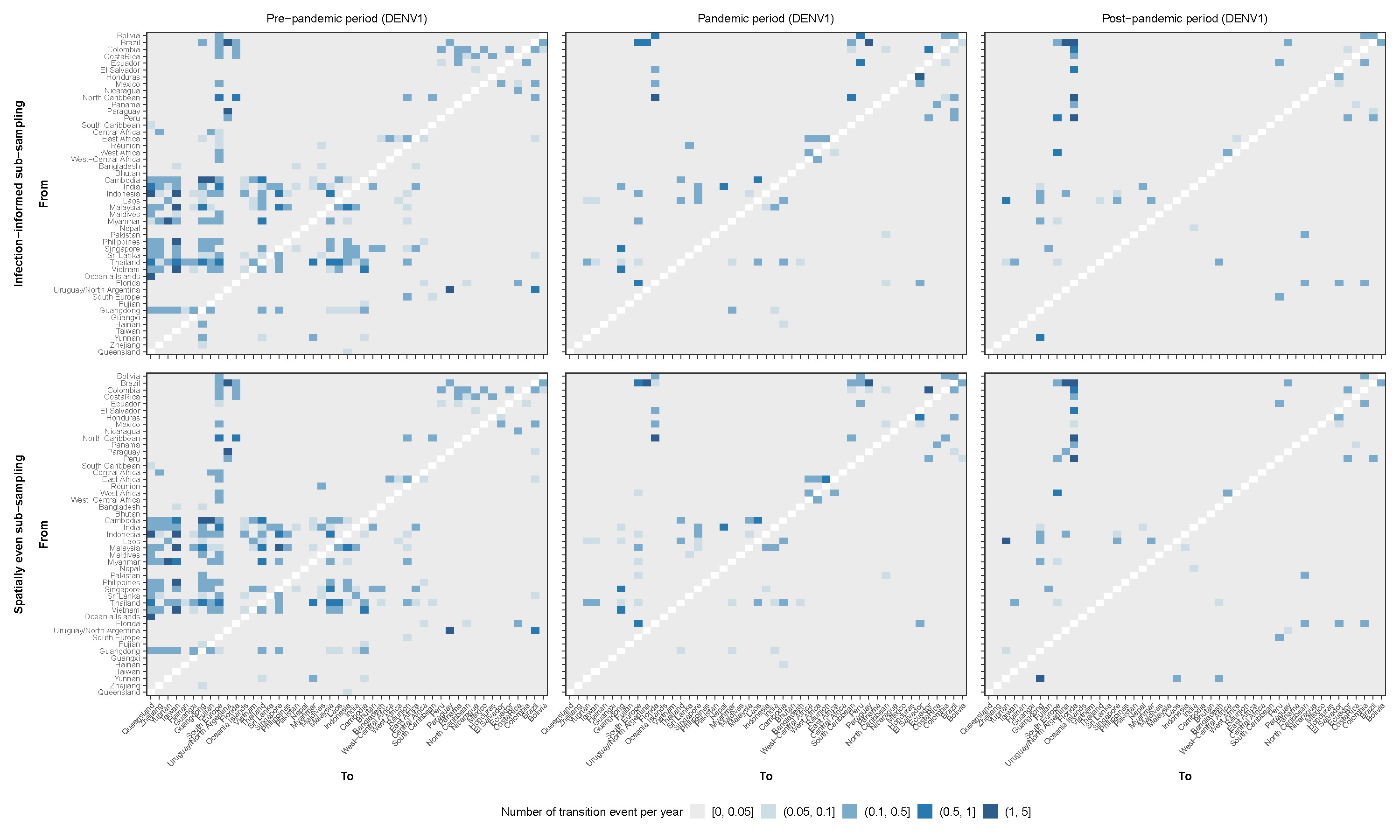
**

**Figure S7. Migration dynamics of DENV1 inferred at sub-location level through three periods.** The first row shows the estimates of annual intensity of Markov jump events (migration events) under infection-informed sub-sampling scheme, and the second row shows that under spatially even sub-sampling scheme.


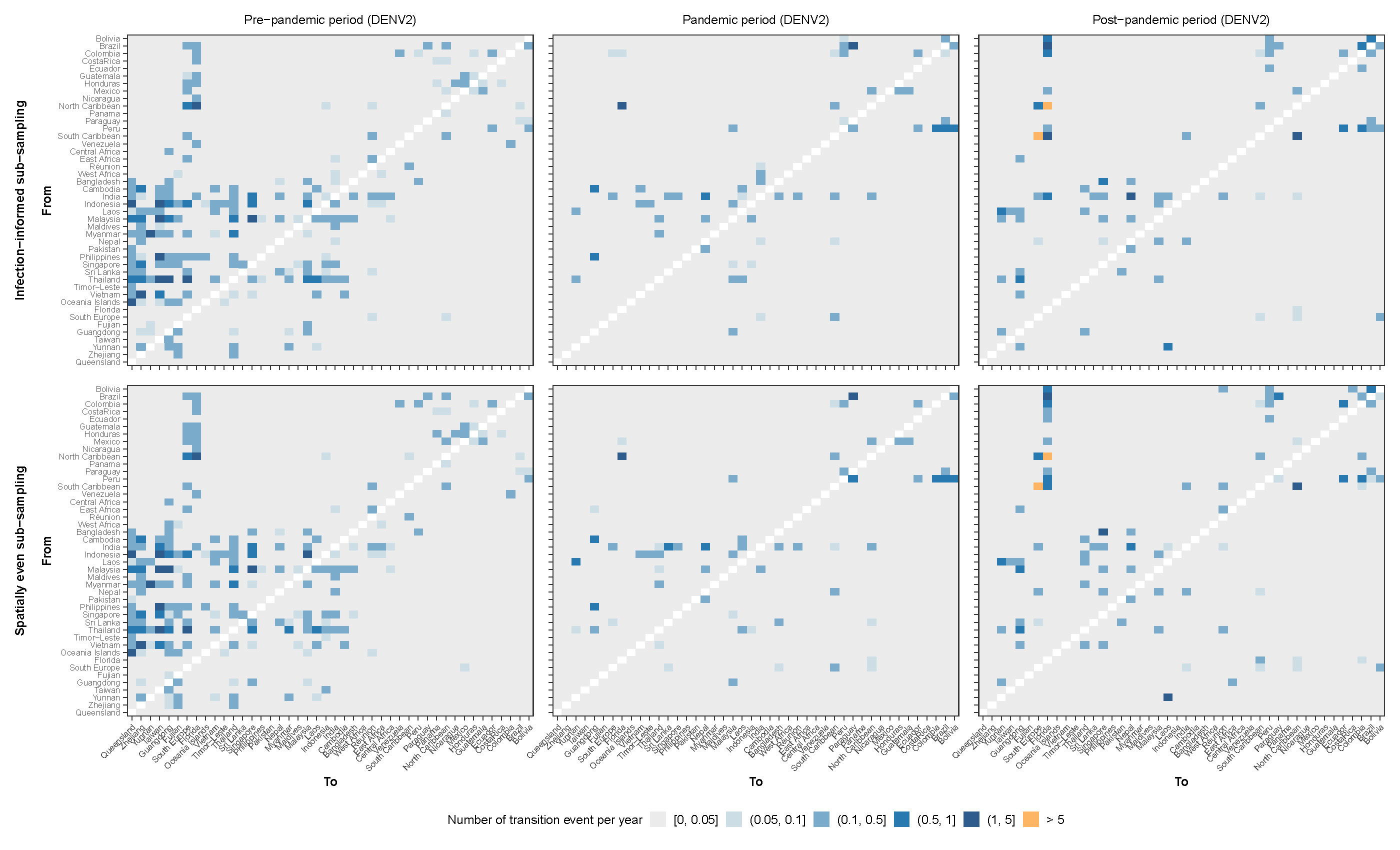


**Figure S8. Migration dynamics of DENV2 inferred at sub-location level through three periods.** The first row shows the estimates of annual intensity of Markov jump events (migration events) under infection-informed sub-sampling scheme, and the second row shows that under spatially even sub-sampling scheme.


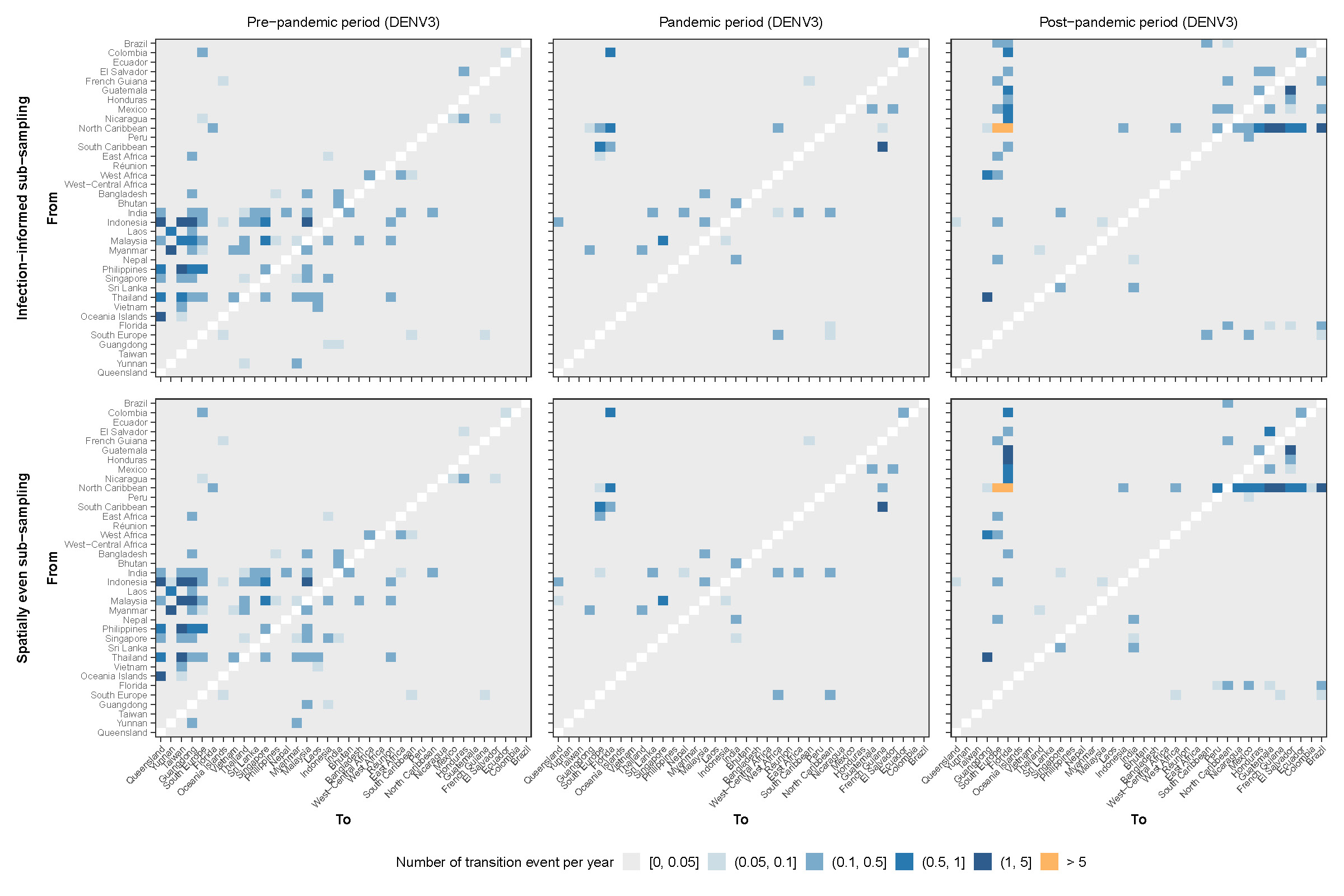


**Figure S9. Migration dynamics of DENV3 inferred at sub-location level through three periods.** The first row shows the estimates of annual intensity of Markov jump events (migration events) under infection-informed sub-sampling scheme, and the second row shows that under spatially even sub-sampling scheme.


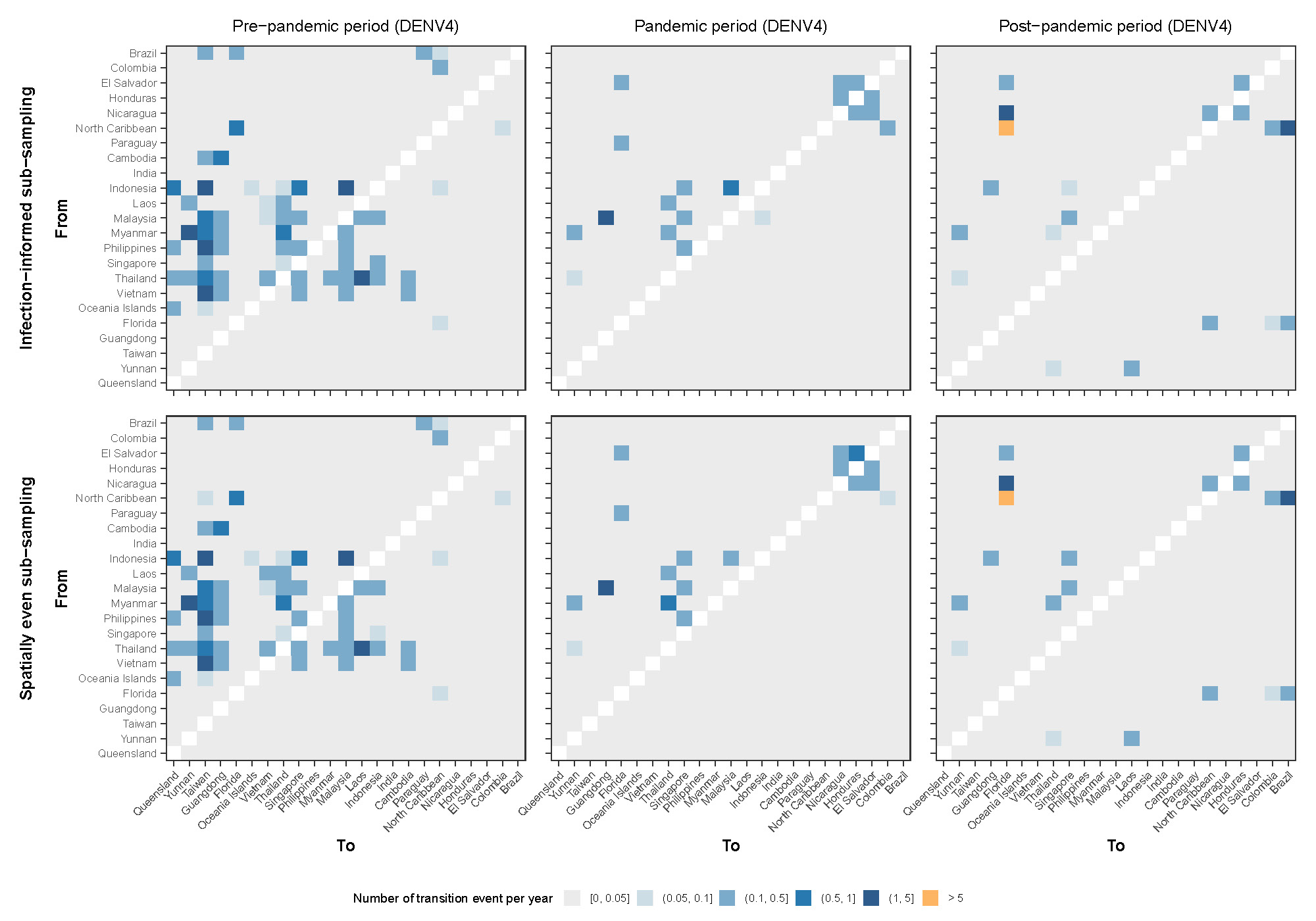


**Figure S10. Migration dynamics of DENV4 inferred at sub-location level through three periods.** The first row shows the estimates of annual intensity of Markov jump events (migration events) under infection-informed sub-sampling scheme, and the second row shows that under spatially even sub-sampling scheme.


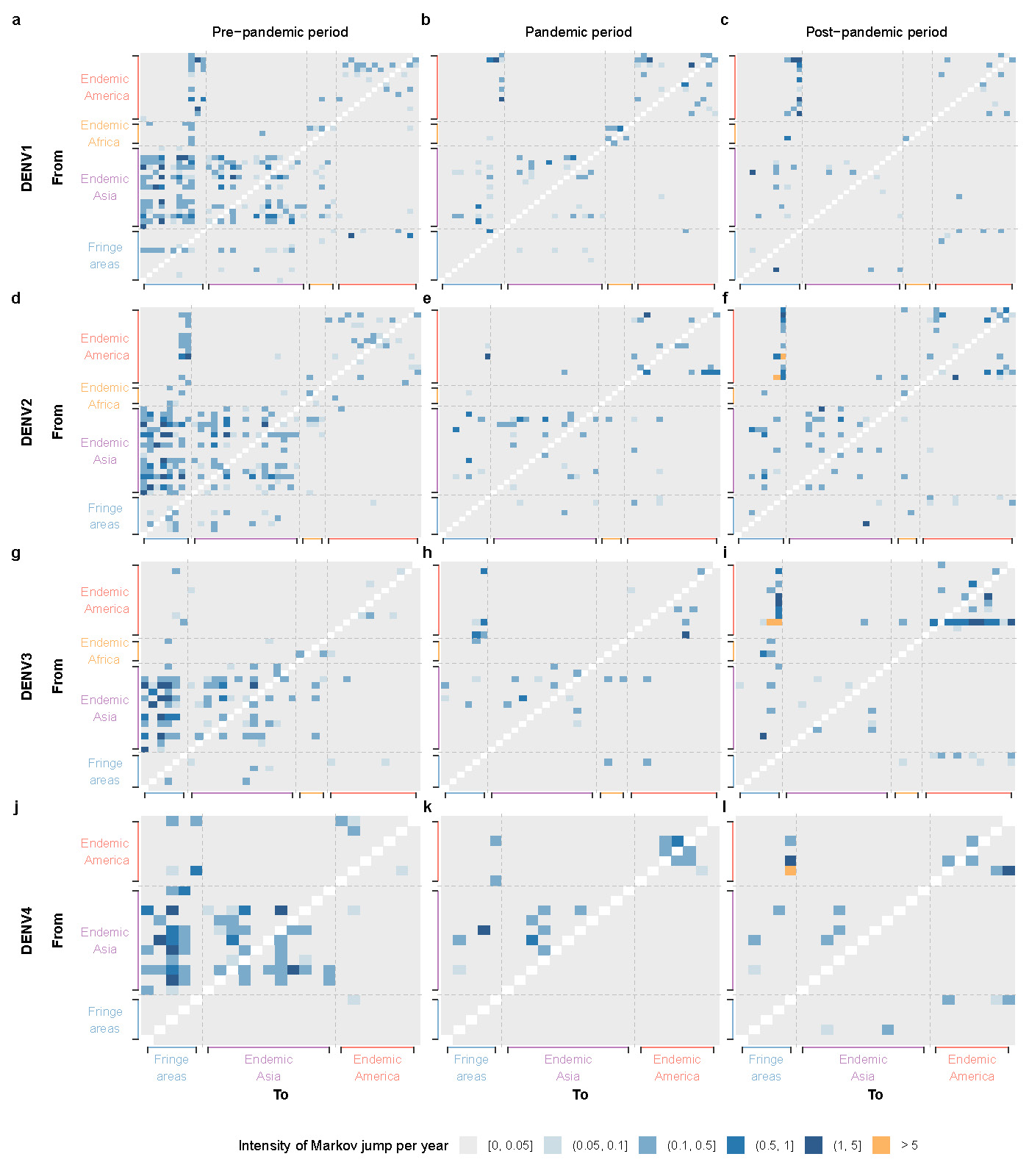


**Figure S11. Global migration dynamics of four DENV serotypes through periods at sub-location level using genetic datasets selected from spatially even sub-sampling scheme. a-l)** Estimates of the number of location transition events (Markov jump) per year between each pair of geographic sub-locations during the pre-pandemic, pandemic, and post-pandemic periods. Analyses are based on the posterior summaries of the Markov jumps under a time-inhomogeneous generalized linear model (GLM)-diffusion phylogeographic model with only air traffic data as the predictor of relative transition rates. The names of sub-locations are not shown in the axis, but are instead categorized within endemic America, endemic Africa, endemic Asia, and fringe areas.


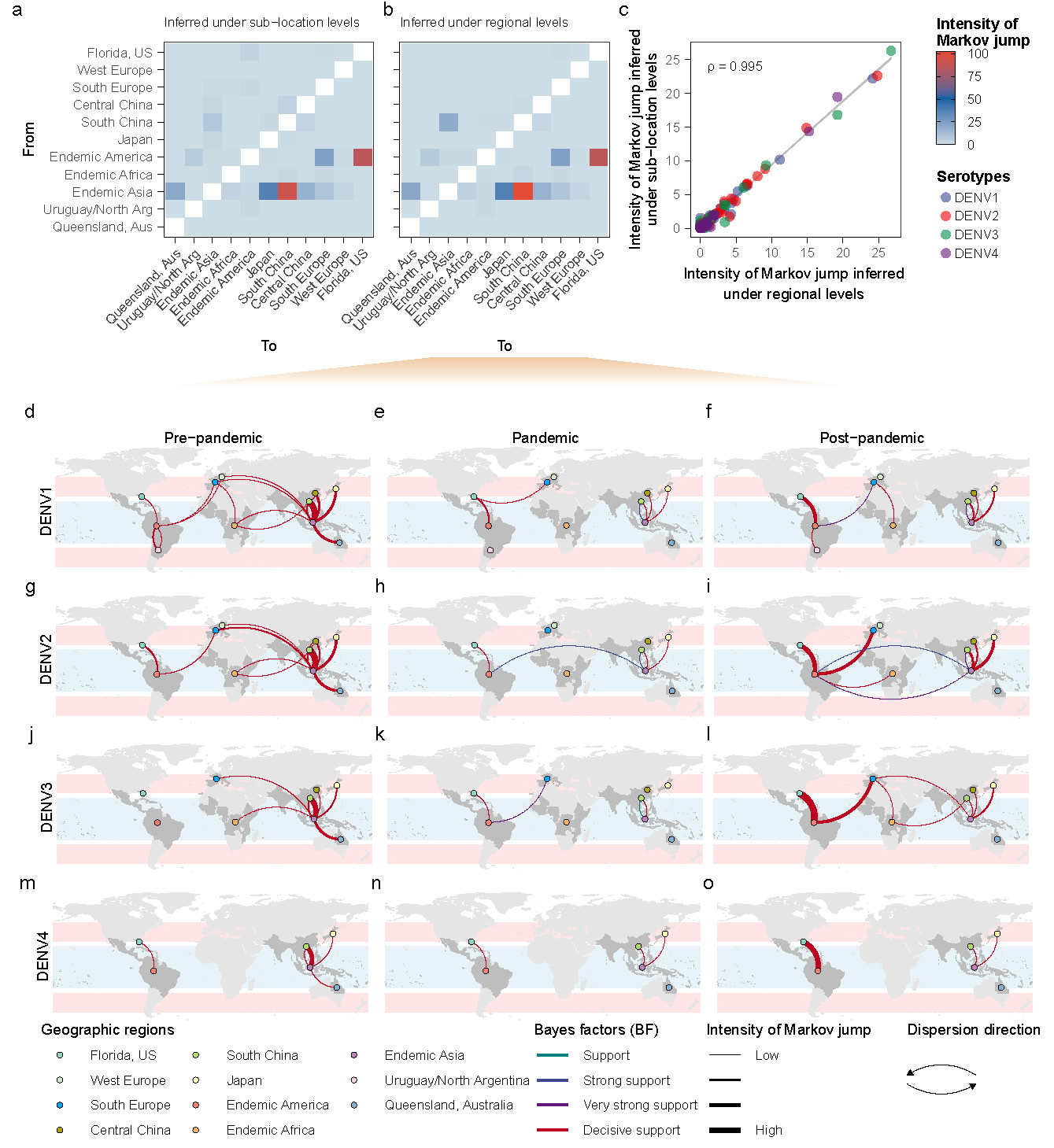


**Figure S12. Global migration routes of dengue virus through three periods at regional level using genetic datasets selected from spatially even sub-sampling scheme. a)** The migration matrix of DENV (four DENV serotypes combined) inferred under the sub-location level**.** The sub-location results are aggregated at the region scale for direct comparison. **b)** The migration matrix of DENV inferred under the regional level**. c)** Association between the serotype- and epoch-specific vectorized transition matrices between each pair of regions, including the Pearson correlation coefficient. **d-o**) Regional-level analyses: estimates of the annual intensity of Markov jump events (migration events) for each DENV serotype between each pair of geographic locations during the pre-pandemic (2010-2019), pandemic (2020-2021), and post-pandemic (2022-2025) periods, respectively. The colored lines represent statistical support for a given viral migration route using Bayes factor (BF), where only migration routes with a BF ≥ 3 are shown. The thicknesses of the curves refer to the annual intensity of Markov jump, where only migration routes with an intensity ≥ 0.5 are shown. The orientation of the curve (convex vs. concave) indicates the directionality of the dispersion (always counter-clockwise). The light-blue and light-red shaded areas represent tropical regions and the two hemispheres adjacent to tropical areas, respectively.


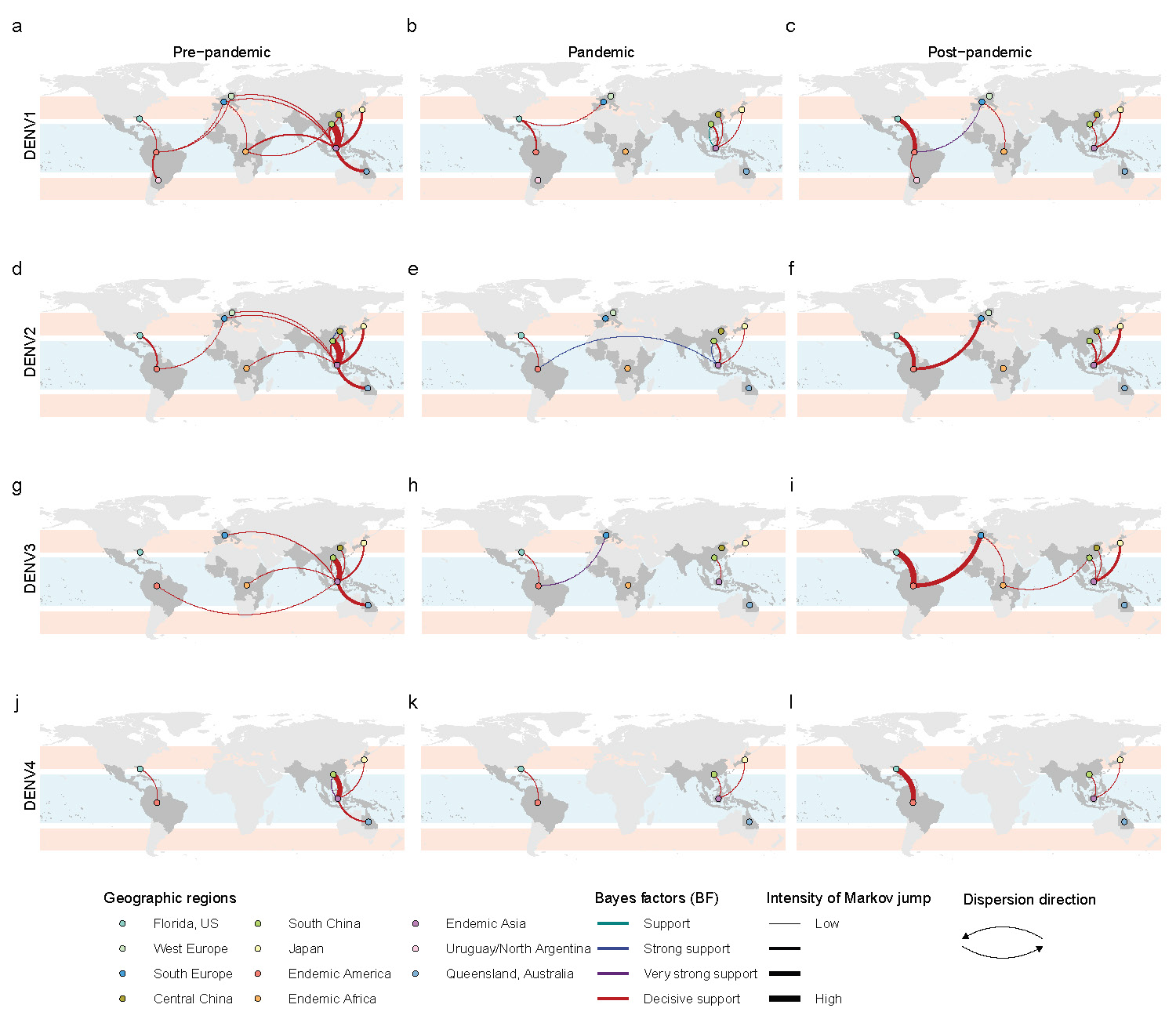


**Figure S13. Estimates of the annual intensity of Markov jump events (migration events) under the down-sampling strategy (a maximum sample size of 50 per sub-location)**. The colored lines represent statistical support for a given viral migration route using Bayes factor (BF), where only migration routes with a BF ≥ 3 are shown. The thicknesses of the curves refer to the annual intensity of Markov jump, where only migration routes with an intensity ≥ 0.5 are shown. The orientation of the curve (convex vs. concave) indicates the directionality of the dispersion (always counter-clockwise). The light-blue and light-red shaded areas represent tropical regions and the two hemispheres adjacent to tropical areas, respectively.


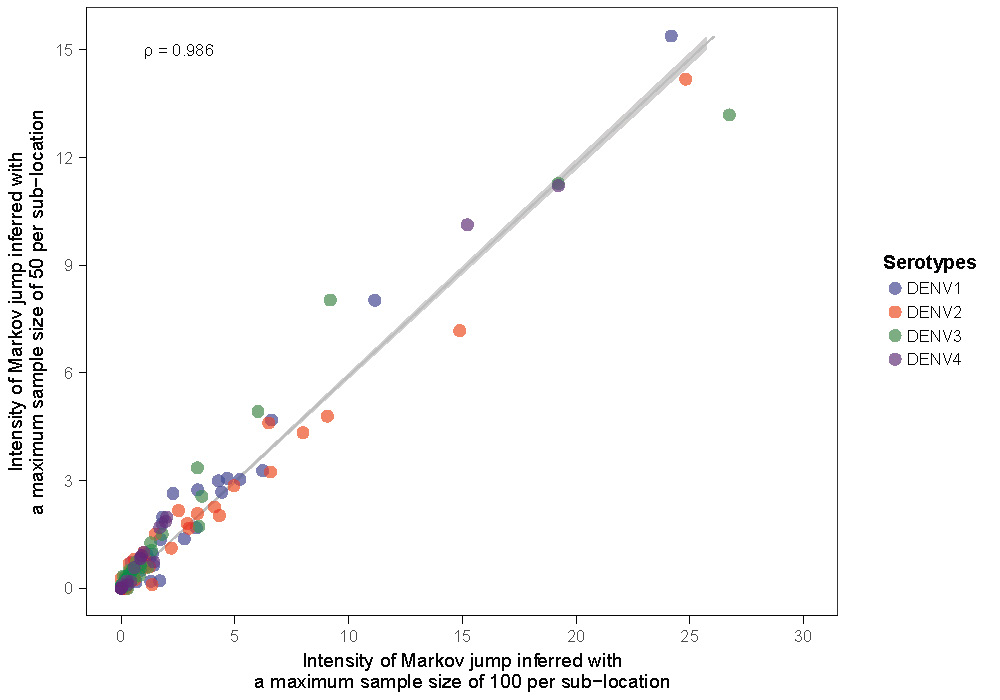


**Figure S14. Association between the serotype- and epoch-specific vectorized transition matrices among each pair of regions when using different sample sizes.**


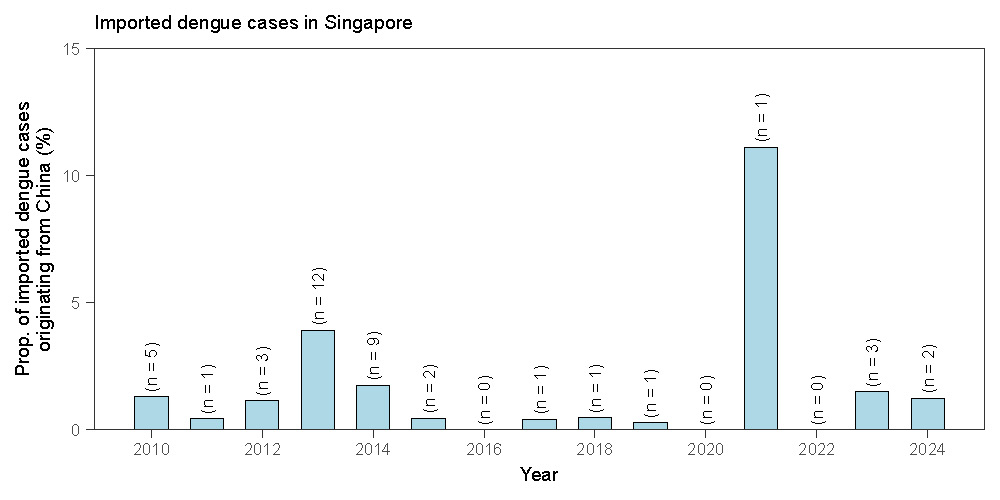


**Figure S15. Annual proportion of imported dengue cases originating from China among all imported cases detected in Singapore**. The numbers in parentheses represent the number of imported dengue cases from China in Singapore. Data were derived from the Communicable Diseases Agency, Singapore (<https://www.cda.gov.sg/resources/>).


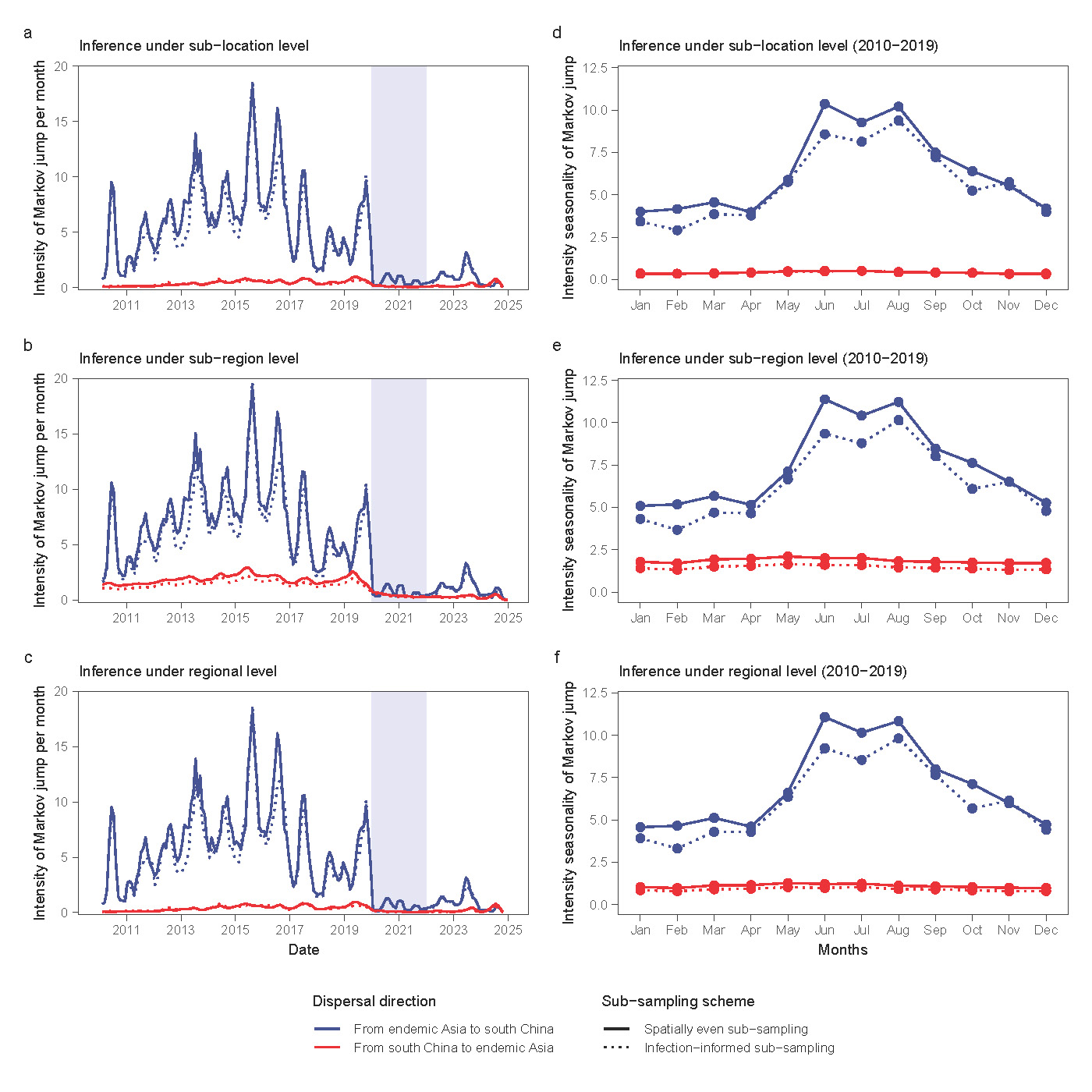


**Figure S16. Posterior estimates of monthly migration intensity of DENV between endemic Asia and South China over time**. **a-c**) Rolling migration intensity of dengue virus (four DENV serotypes combined) between endemic Asia and south China inferred under different spatial resolutions. For each resolution, comparisons are made between infection-informed (dashed line) and spatially even (solid line) sub-sampling schemes. The light-purple shaded area represents the COVID-19 pandemic period, defined as January 2020 to December 2021. **d-f**) The mean seasonal patterns of migration intensity of dengue virus in two dispersal directions. Mean seasonal patterns were calculated by aggregating migration intensity from 2010 to 2019 into 12 monthly bins and taking the average in each month.


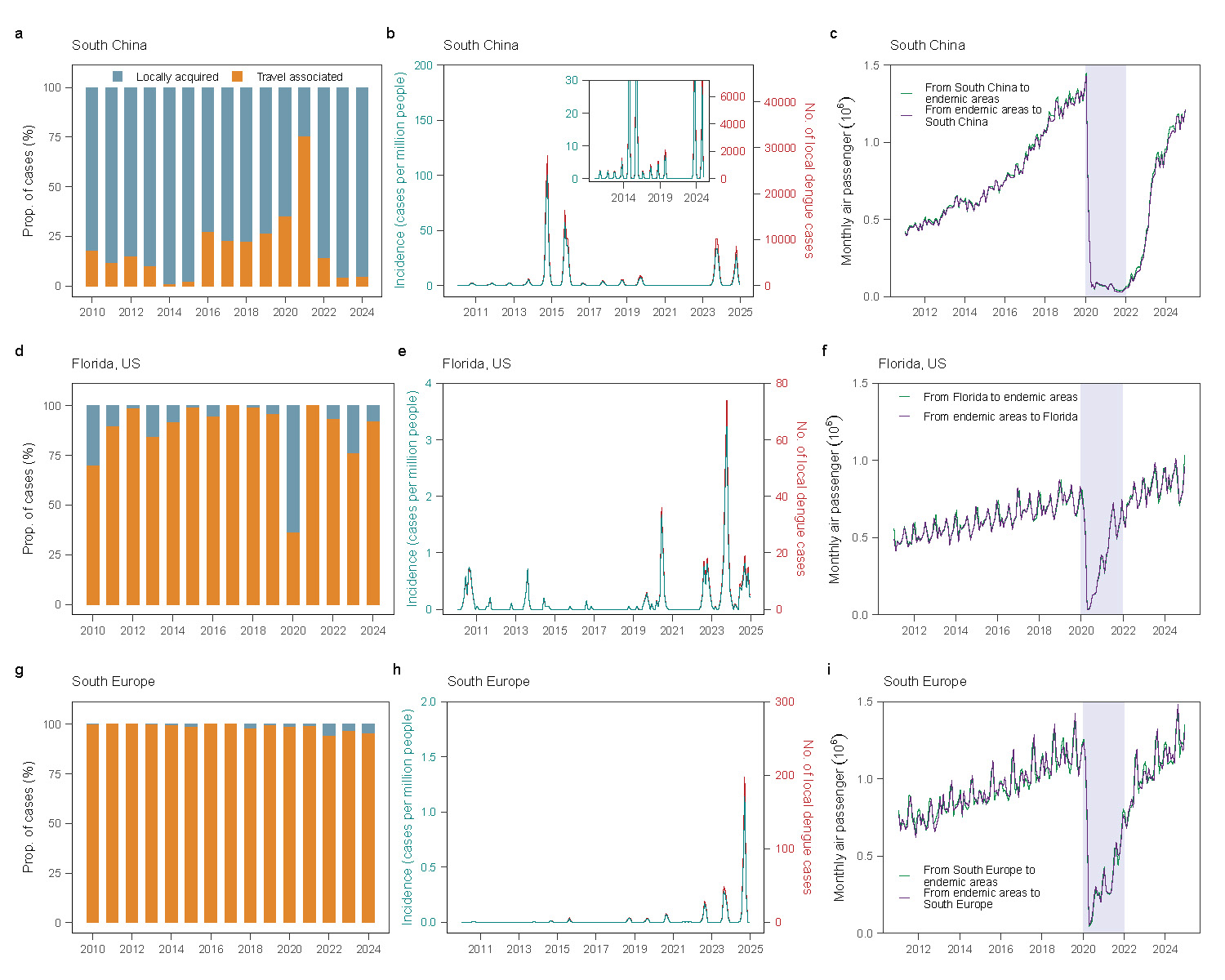


**Figure S17. Comparison of dengue epidemics among three fringe areas on the fringe of endemic transmission.** **a**) Annual proportion of locally acquired and travel associated cases in South China (only included data from Guangdong, Yunnan, Zhejiang, Fujian, and Taiwan provinces due to data availability). **b**) The monthly incidence (green line) and number (red line, which is virtually indistinguishable from the incidence as they are highly consistent) of locally acquired cases in South China (Guangdong, Yunnan, Zhejiang, Fujian, and Taiwan). **c**) Monthly air passenger between endemic areas and South China (Guangdong, Yunnan, Zhejiang, Fujian, and Taiwan). **d-f**) Same as (**a-c**) but for Florida, US. **g-i**) Same as (**a-c**) but for South Europe (France, Italy, Spain).

**
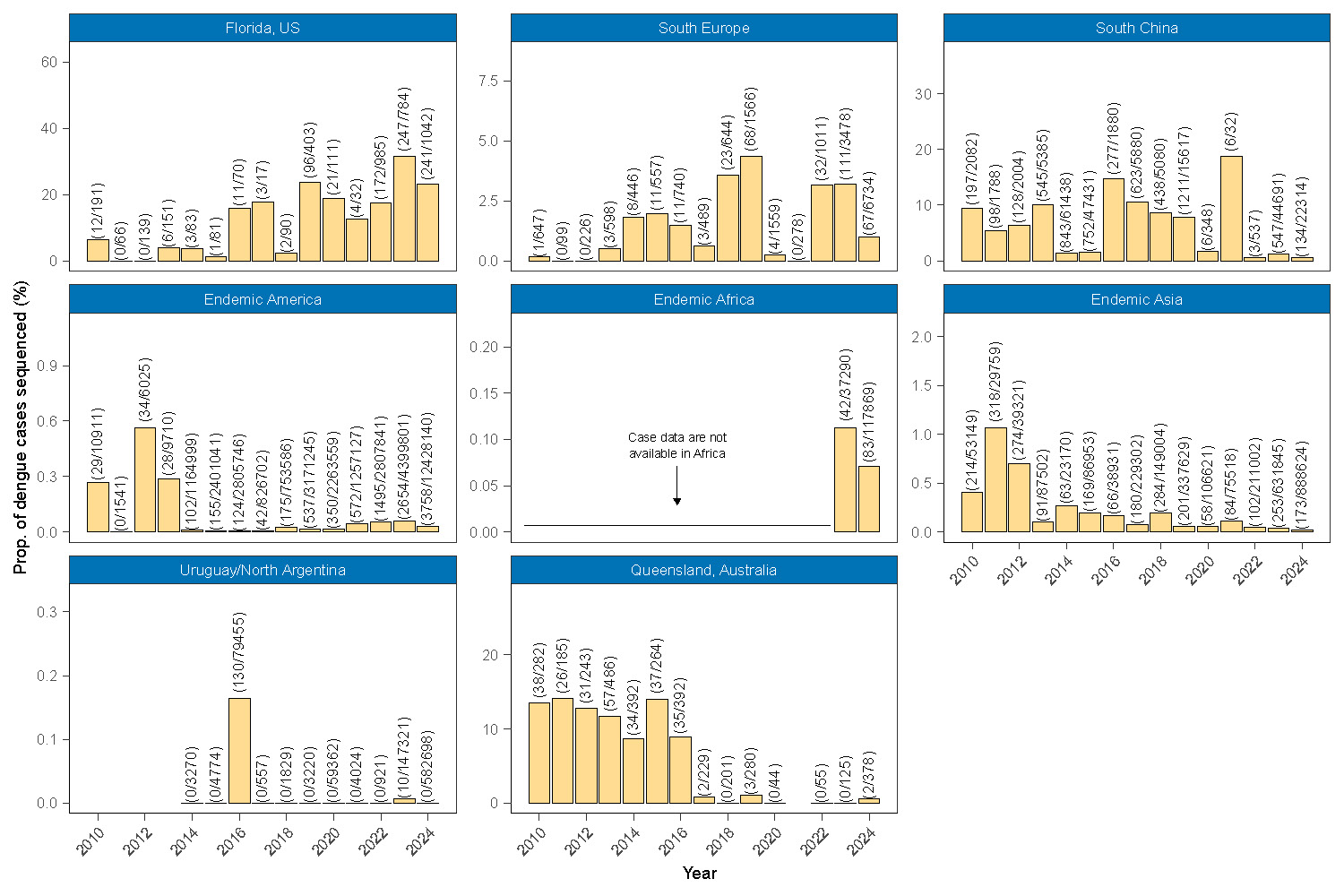
**

**Figure S18. The** **annual percentages of reported DENV cases sequenced for each geographic region.** Sequencing rates cannot be calculated in some years due to limited availability of case data, such as in endemic Africa from 2010 to 2022. The numerator on the bar chart represents the number of sequences publicly available this year, and the denominator represents the number of cases reported during the same year. The y-axis range differs across panels.


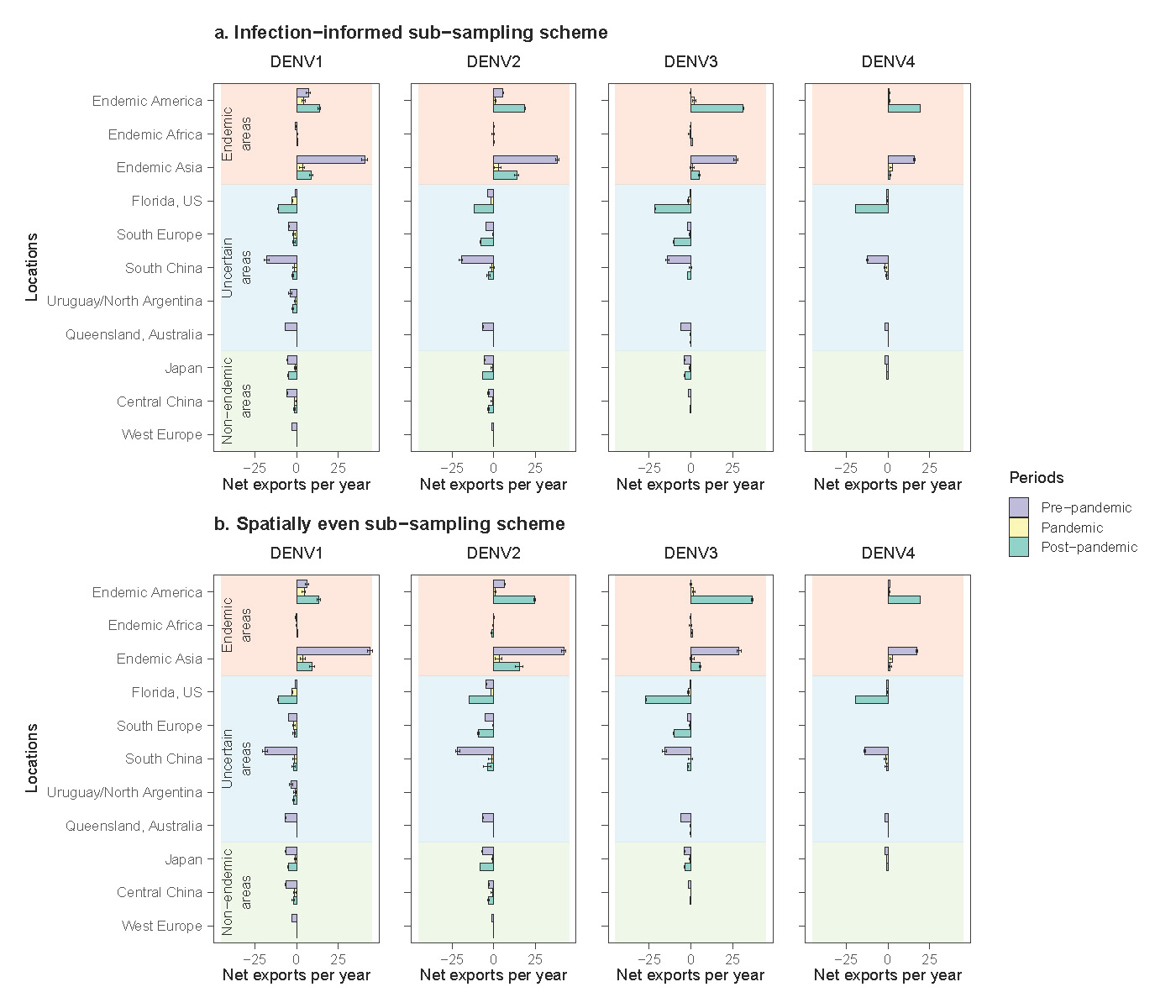


**Figure S19. The period-specific net export dynamic of dengue virus. a)** Inference using datasets from infection-informed sub-sampling scheme; **b)** Inference using datasets from spatially even sub-sampling scheme. The average annual number of net lineage export events were defined as the export event count minus import event count per year. Posterior mean and 90% HPD intervals are reported.


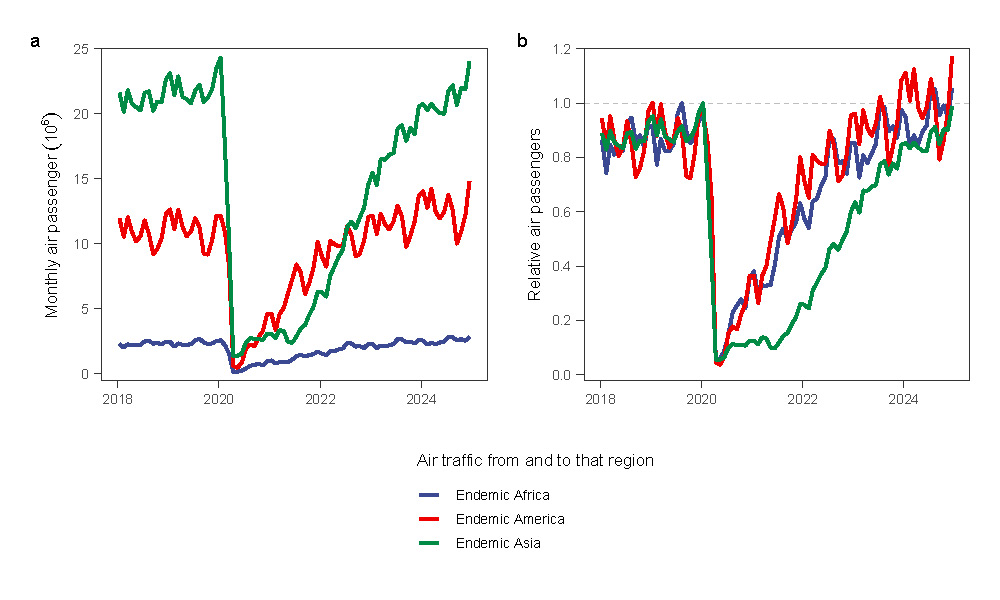


**Figure S20. The global air traffic from and to each endemic region from 2018 to 2024. a)** Monthly air passenger traffic from and to each endemic region. **b)** Relative air traffic from and to each region over time, calculated by dividing the numbers by the maximum value of each region during the pre-pandemic period.


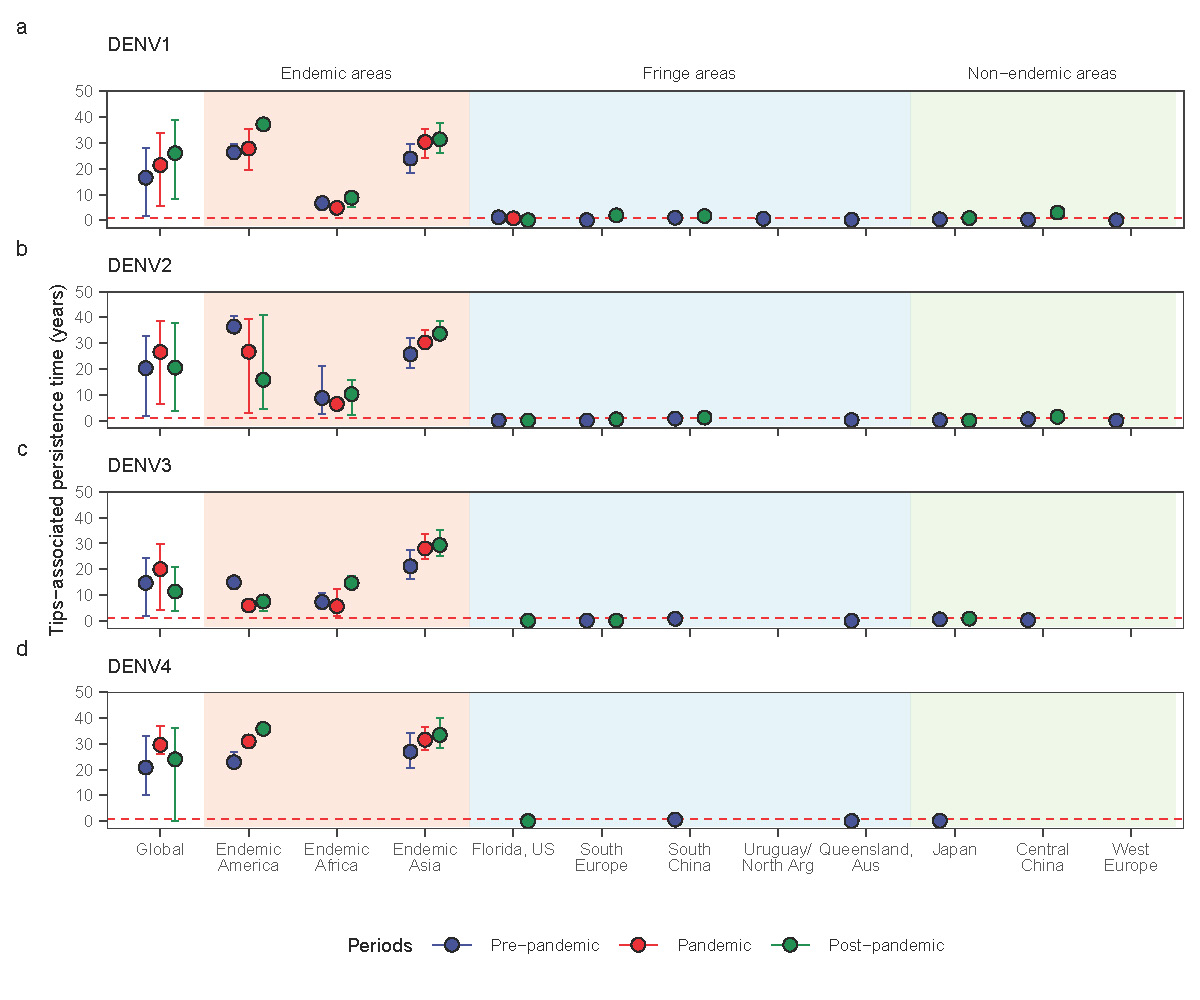


**Figure S21. Tip-associated persistence of dengue virus time across periods.** Tip-associated persistence time represents the time for tips in the phylogeny to leave its current sampling location when moving backwards in time. Some locations lack estimates of persistence time due to the low number of sampled tips available at that time period. Points refer to mean persistence time and error bars represent the interquartile range.


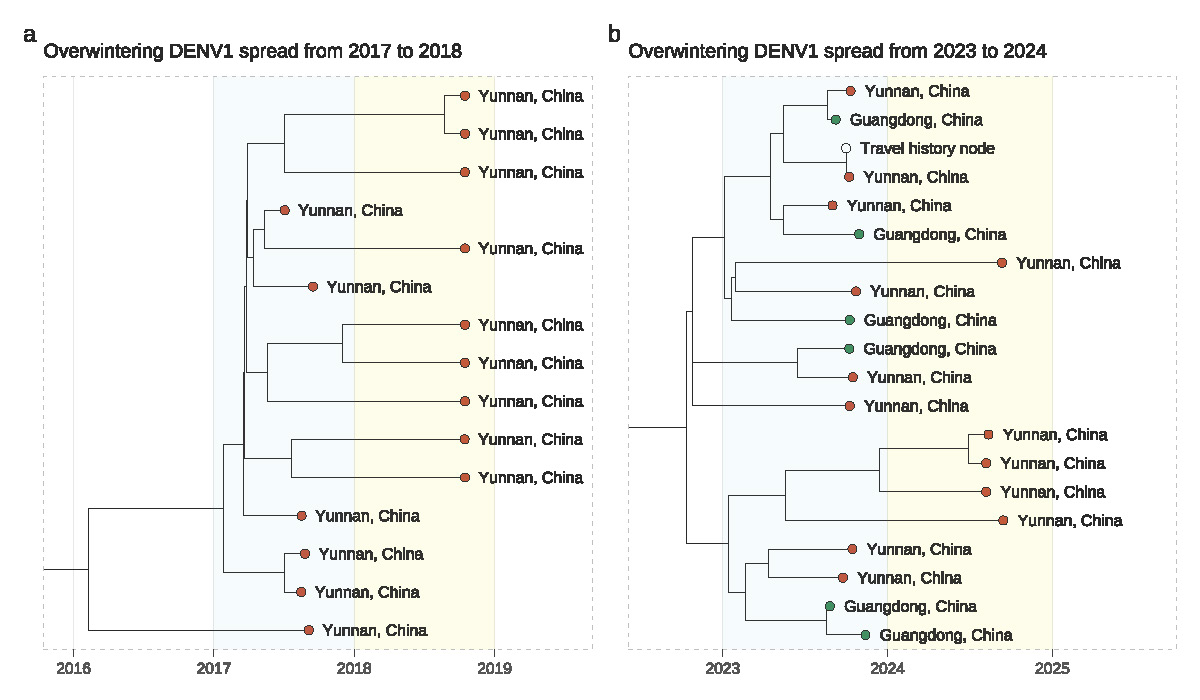


**Figure S22. The phylogenetic evidence of overwintering DENV1 spread in South China**. Tips are colored by the sampling locations of sequences.


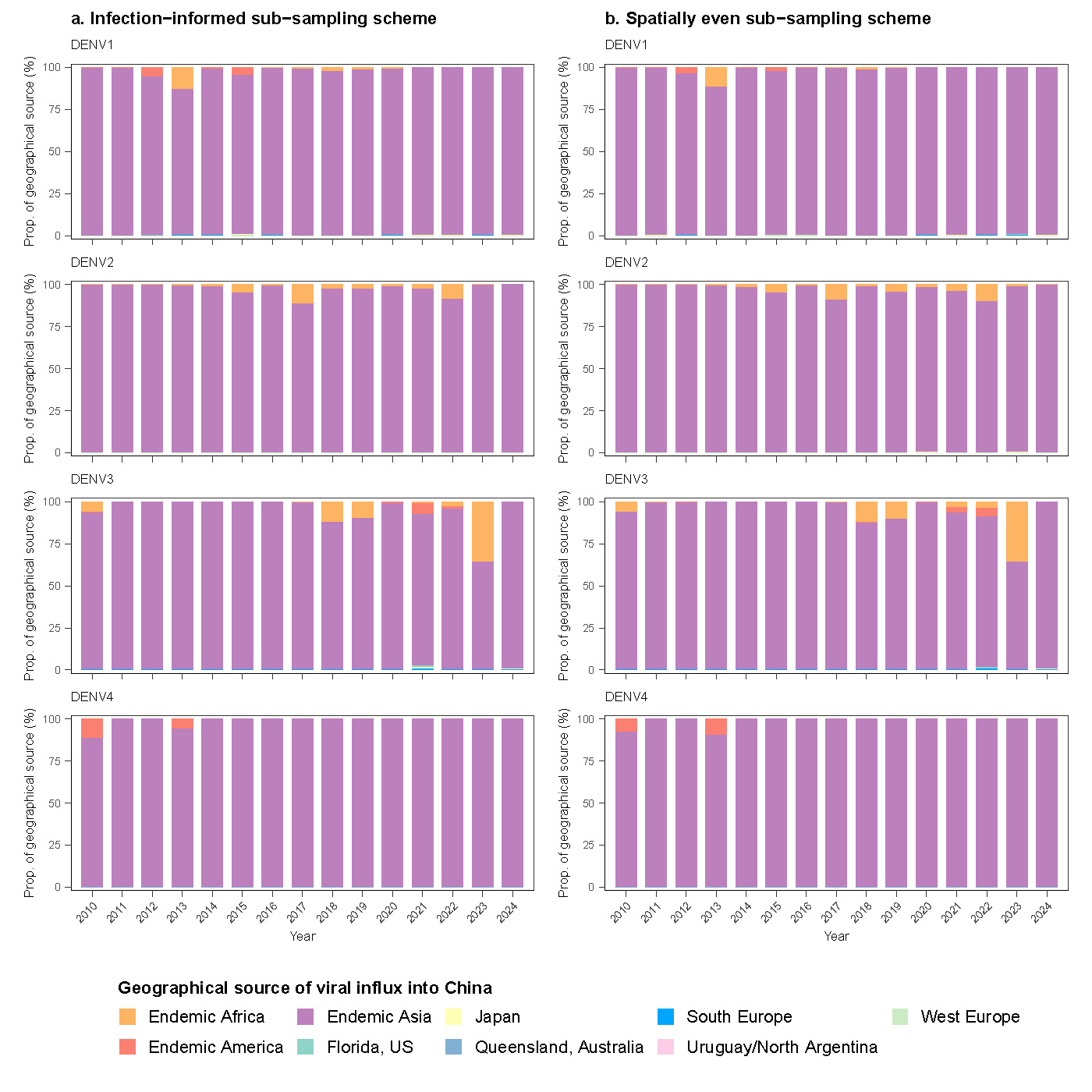


**Figure S23.** **Geographical source of DENV viral influx into China**. **a)** Inference using datasets from infection-informed sub-sampling scheme; **b)** Inference using datasets from spatially even sub-sampling scheme. The results are based on posterior summaries of the Markov jumps from the regional-level analyses.


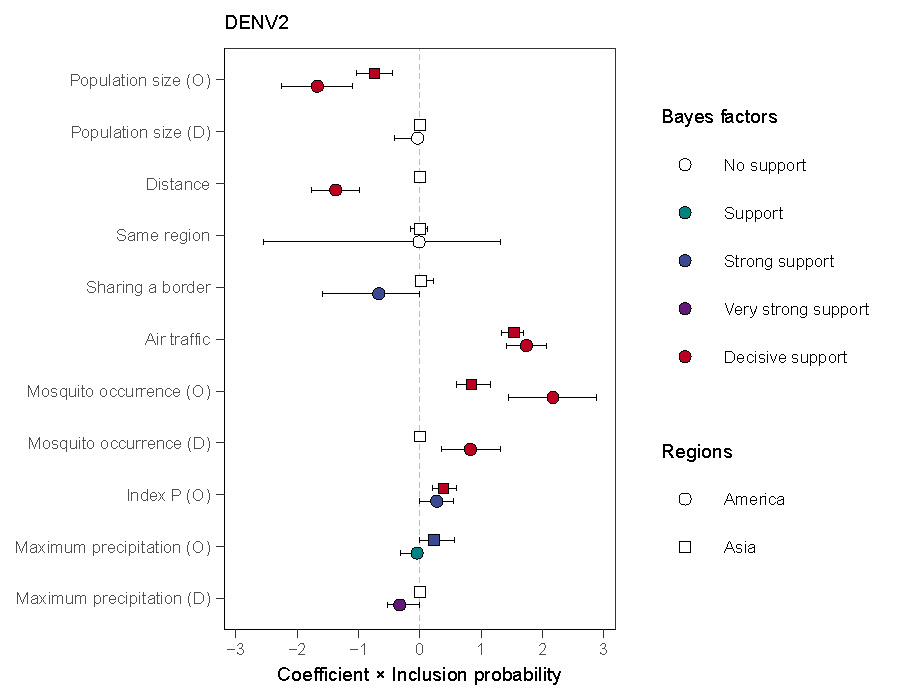


**Figure S24. Predictors of DENV2 spread across Asia and the Americas**. The posterior summaries of the product (reported as log effect size) of the constant through-time predictor inclusion probability and the log predictor coefficient for each serotype were shown. The colored points represent statistical support using Bayes factor (BF). Points and ranges represent the posterior mean and 95% HPD intervals, respectively. Location specific predictors were included as both origin (O) and destination (D) predictors of the pairwise transition rates except for index P.


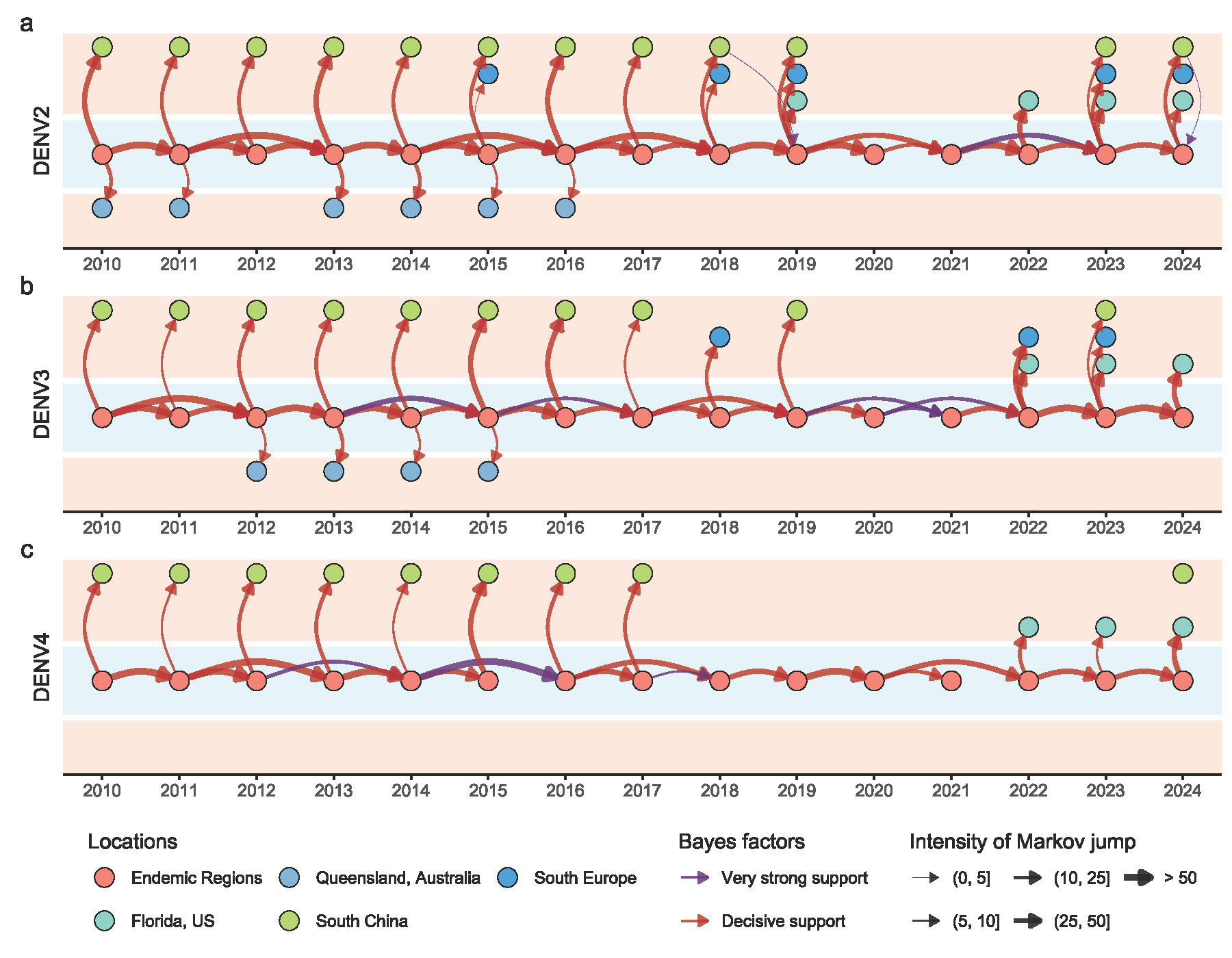


**Figure 25. Overwintering migration patterns among geographic locations inferred under a fixed-indicator and fixed-root model.** **a-c)** Estimates of multiple-year migration intensity within and between geographic locations for each serotype respectively. The model configuration proved impractical for achieving adequate mixing in the DENV1 analysis, and thus results for DENV2–4 are only shown. Some locations in the northern and southern hemisphere lack points due to limited availability of corresponding genetic data. The colored lines represent statistical support for a given viral migration route using Bayes factor (BF), where only migration routes with a BF ≥ 100 are shown. The thicknesses of the curves refer to the intensity of the Markov jump event.

# Supplementary tables

**Tables S1. List of countries/territories in each endemic region included in this study.**

| **Regions** | **Countries/territories** |
| --- | --- |
| **Endemic Africa** | |
| Central Africa | Angola, Democratic Republic of the Congo |
| East Africa | Djibouti, Eritrea, Ethiopia, Kenya, Mozambique, Mayotte, Réunion, Somalia, Seychelles, Tanzania |
| West-Central Africa | Cameroon, Gabon, Nigeria |
| West Africa | Burkina Faso, Côte d'Ivoire, Ghana, Guinea, The Gambia, Mali, Senegal, Sao Tome and Principe |
| **Endemic America** | |
| Caribbean | Saint Barthelemy, Barbados, Cuba, Dominica, Dominican Republic, Guadeloupe, Grenada, Haiti, Jamaica, Saint Martin (French part), Martinique, Puerto Rico, Trinidad and Tobago, Saint Vincent and the Grenadines |
| Central America | Costa Rica, Guatemala, Honduras, Mexico, Nicaragua, Panama, El Salvador |
| South America | Bolivia, Brazil, Colombia, Ecuador, French Guiana, Peru, Paraguay, Venezuela |
| **Endemic Asia** | |
| South Asia | Bangladesh, Bhutan, India, Sri Lanka, Maldives, Nepal, Pakistan |
| Southeast Asia | Indonesia, Cambodia, Laos, Myanmar, Malaysia, Philippines, Singapore, Thailand, Timor-Leste, Vietnam |
| Oceania Islands | American Samoa, Cook Islands, Fiji, Micronesia, Kiribati, Marshall Islands, New Caledonia, Niue, Nauru, Papua New Guinea, French Polynesia, Solomon Islands, Tonga, Tuvalu, Vanuatu, Wallis and Futuna, Samoa |

** These regions are defined based on the United Nations geo-scheme.*

**Table S2. List of endemic countries/territories in Africa classified by WHO or the United States (US) CDC.**

| **Sub-regions** | **Countries** | **US CDC (2010)^42^** | **US CDC (2012)^42^** | **WHO (2018)^1^** | **US CDC (2025)^2^** |
| --- | --- | --- | --- | --- | --- |
| Central Africa | Angola | Endemic | Endemic | / | / |
|  | DR Congo | Endemic | Endemic | / | / |
| East Africa | Kenya | Endemic | Endemic | Endemic | Endemic |
|  | Ethiopia | Endemic | Endemic | Endemic | Endemic |
|  | Somalia | Endemic | Endemic | Endemic | Endemic |
|  | Tanzania | Endemic | Endemic | Endemic | Endemic |
|  | Mozambique | Endemic | Endemic | / | / |
|  | Eritrea | Endemic | Endemic | / | Endemic |
|  | Seychelles | / | Endemic | / | Endemic |
|  | Djibouti | Endemic | Endemic | Endemic | / |
|  | Mayotte | / | Endemic | / | Endemic |
|  | Réunion | / | / | Endemic | Endemic |
| West-Central Africa | Cameroon | Endemic | Endemic | Endemic | Endemic |
|  | Gabon | / | Endemic | / | Endemic |
|  | Nigeria | Endemic | Endemic | Endemic | / |
| West Africa | Burkina Faso | Endemic | Endemic | / | Endemic |
|  | Côte d'Ivoire | / | Endemic | Endemic | Endemic |
|  | Sao Tome and Principe | / | Endemic | / | / |
|  | Senegal | Endemic | Endemic | Endemic | Endemic |
|  | Mali | / | Endemic | Endemic | / |
|  | Ghana | Endemic | / | / | / |
|  | Guinea | Endemic | Endemic | Endemic | / |
|  | The Gambia | Endemic | / | Endemic | / |

* *Endemic risk classification from US CDC adopts the methodology described in Jentes, et al.^43^, where areas with more than 10 dengue cases reported in at least three distinct years over the most recent 10-year period would be assumed as endemic areas.*

**Table S3. Quantitative metrics used for defining fringe areas.**

| **Areas** | **Proportion of local cases (%) with the number of local cases (n)** | **Annual duration of local transmission (median)** |
| --- | --- | --- |
| Florida, US | 2010: 30.4% (n = 58)  2020: 64.0% (n = 71)  2022: 6.8% (n = 67)  2023: 24.1% (n = 189)  2024: 8.0% (n = 83) | 7 months |
| South Europe | 2022: 5.9% (n = 60)  2023: 4.9% (n = 327) | 4.5 months |
| South China | 2010: 82.3% (n = 1,714)  2011: 88.4% (n = 1,581)  2012: 85.5% (n = 1,713)  2013: 90.2% (n = 4,860)  2014: 99.3% (n = 61,020)  2015: 98.1% (n = 46,534)  2016: 73.1% (n = 1,918)  2017: 77.4% (n = 4,554)  2018: 77.9% (n = 3,961)  2019: 73.9% (n = 11,574)  2020: 65.0% (n = 316)  2022: 86.1% (n = 538)  2023: 95.9% (n = 43,120)  2024: 95.5% (n = 21,419) | 5 months |
| Uruguay/North Argentina | 2024 (Uruguay): 62.5% (n = 712)  2023 (North Argentina): 98.7% (n = 130,287)  2024 (North Argentina): 97.6% (n = 1,188) * | 6.5 months |
| Queensland, Australia | 2013: 12.8% (n = 236)  2014: 10.9% (n = 188) | 7 months |

* Only data from weeks 31 through 52 of 2024 are publicly available.

**Table S4. Proportion of DENV sequences having information of travel history by each location.**

| **Locations** | **Total** | **No. of seqs having travel history** | **No. of seqs from local cases** | **Unknown** | **Literature sources of travel history** |
| --- | --- | --- | --- | --- | --- |
| Florida, US | 844 | 706 (83.6%) | 95 (11.3%) | 43 (5.1%) | ^44-46^ |
| South Europe | 342 | 217 (63.5%) | / | 125 (36.5%) | France: ^47^  Spain: ^48-50^  Italy: ^51,52^ |
| West Europe | 57 | 55 (96.5%) | / | 2 (3.5%) | Switzerland: ^49^  Belgium: ^49^  Germany: ^53^ |
| Central China | 392 | 137 (34.9%) | 112 (28.6%) | 143 (36.5%) | Shandong: ^54^  Hunan: ^55,56^  Jiangsu: ^57^  Hubei: ^58^  Henan: ^59-62^  Shanghai: ^63,64^  Sichuan: ^65,66^ |
| South China | 6,089 | 1,551 (25.5%) | 1,915 (31.5%) | 2,623 (43.1%) | Guangdong: ^67-79^  Yunnan: ^68,80-96^  Fujian: ^68,97-100^  Hainan: ^101^  Guangxi: ^102,103^  Zhejiang: ^58,104-108^ |
| Japan | 298 | 178 (59.7%) | / | 120 (40.3%) |  |
| Uruguay/North Argentina | 147 | 40 (27.2%) | / | 107 (72.8%) | ^109,110^ |
| Queensland, Australia | 265 | 207 (78.1%) | / | 58 (21.9%) | ^111^ |

**Table S5. Travel history information of DENV sequences in each location.**

| **Travel history** |  | | **Locations** | | | | | | |
| --- | --- | --- | --- | --- | --- | --- | --- | --- | --- |
|  | Florida, US | South Europe | | West Europe | Central China | South China | Japan | Uruguay/North Argentina | Queensland, Australia |
| Endemic Africa | / | 2.8 (6/217) | | 5.5 (3/55) | 2.9 (4/137) | 1.4 (22/1,551) | 2.2  (4/178) | / | / |
| Endemic America | 97.7 (690/706) | 54.4 (118/217) | | 21.8 (12/55) | / | 0.5  (7/1,551) | 1.7  (3/178) | 97.5  (39/40) | 0.5  (1/207) |
| Endemic Asia | 1.6 (11/706) | 36.4 (79/217) | | 72.7 (40/55) | 78.8 (108/137) | 94.3 (1463/1,551) | 94.4 (168/178) | / | 99.5 (206/207) |
| Others | 0.1 (1/706) | 0.9 (2/217) | | / | 6.6 (9/137) | 1.8 (28/1,551) | 1.7  (3/178) | 2.5  (1/40) | / |
| Unclear | 0.6 (4/706) | 5.5 (12/217) | | / | 11.7 (16/137) | 2.0 (31/1,551) | / | / | / |

# References

1. World Health Organization. Ending the neglect to attain the sustainable development goals: a road map for neglected tropical diseases 2021–2030. Geneva: World Health Organization; 2020.

2. Liliana S-G, Laura EA, Gabriela P-B. CDC Yellow Book: Health Information for International Travel. 2026.

3. Sohail A, Anders KL, McGuinness SL, Leder K. The epidemiology of imported and locally acquired dengue in Australia, 2012–2022. *Journal of travel medicine* 2024; **31**(2): taae014.

4. World Health Organization. WHO Global Dengue Dashboard. 2026. <https://worldhealthorg.shinyapps.io/dengue_global/>.

5. Wallau GL. Arbovirus researchers unite: expanding genomic surveillance for an urgent global need. *The Lancet Global health* 2023; **11**(10): e1501-e2.

6. Brister JR, Ako-Adjei D, Bao Y, Blinkova O. NCBI viral genomes resource. *Nucleic acids research* 2015; **43**(D1): D571-7.

7. Aksamentov. I, Roemer. C, Hodcroft. EB, Neher. RA. Nextclade: clade assignment, mutation calling and quality control for viral genomes. *Journal of Open Source Software* 2021; **6**: 3773.

8. Hill V, Cleemput S, Pereira JS, et al. A new lineage nomenclature to aid genomic surveillance of dengue virus. *PLoS biology* 2024; **22**(9): e3002834.

9. Katoh K, Standley DM. MAFFT Multiple Sequence Alignment Software Version 7: Improvements in Performance and Usability. *Molecular Biology and Evolution* 2013; **30**(4): 772-80.

10. Hadfield J, Megill C, Bell SM, et al. Nextstrain: real-time tracking of pathogen evolution. *Bioinformatics* 2018; **34**(23): 4121-3.

11. Chen Z, Tsui JLH, Gutierrez B, et al. COVID-19 pandemic interventions reshaped the global dispersal of seasonal influenza viruses. *Science* 2024; **386**(6722): eadq3003.

12. Lemey P, Hong SL, Hill V, et al. Accommodating individual travel history and unsampled diversity in Bayesian phylogeographic inference of SARS-CoV-2. *Nature communications* 2020; **11**(1): 5110.

13. Grubaugh ND, Saraf S, Gangavarapu K, et al. Travel Surveillance and Genomics Uncover a Hidden Zika Outbreak during the Waning Epidemic. *Cell* 2019; **178**(5): 1057-71.e11.

14. Nakase T, Giovanetti M, Obolski U, Lourenço J. Global transmission suitability maps for dengue virus transmitted by Aedes aegypti from 1981 to 2019. *Scientific Data* 2023; **10**(1): 275.

15. Hersbach H, Bell B, Berrisford P, et al. The ERA5 global reanalysis. *Quarterly Journal of the Royal Meteorological Society* 2020; **146**(730): 1999-2049.

16. Kraemer MU, Sinka ME, Duda KA, et al. The global distribution of the arbovirus vectors Aedes aegypti and Ae. albopictus. *eLife* 2015; **4**: e08347.

17. Price MN, Dehal PS, Arkin AP. FastTree 2--approximately maximum-likelihood trees for large alignments. *PloS one* 2010; **5**(3): e9490.

18. Martin DP, Varsani A, Roumagnac P, et al. RDP5: a computer program for analyzing recombination in, and removing signals of recombination from, nucleotide sequence datasets. *Virus evolution* 2021; **7**(1): veaa087.

19. Martin D, Rybicki E. RDP: detection of recombination amongst aligned sequences. *Bioinformatics* 2000; **16**(6): 562-3.

20. Padidam M, Sawyer S, Fauquet CM. Possible emergence of new geminiviruses by frequent recombination. *Virology* 1999; **265**(2): 218-25.

21. Martin DP, Posada D, Crandall KA, Williamson C. A modified bootscan algorithm for automated identification of recombinant sequences and recombination breakpoints. *AIDS research and human retroviruses* 2005; **21**(1): 98-102.

22. Smith JM. Analyzing the mosaic structure of genes. *Journal of molecular evolution* 1992; **34**(2): 126-9.

23. Posada D, Crandall KA. Evaluation of methods for detecting recombination from DNA sequences: computer simulations. *Proceedings of the National Academy of Sciences of the United States of America* 2001; **98**(24): 13757-62.

24. Gibbs MJ, Armstrong JS, Gibbs AJ. Sister-scanning: a Monte Carlo procedure for assessing signals in recombinant sequences. *Bioinformatics* 2000; **16**(7): 573-82.

25. Lam HM, Ratmann O, Boni MF. Improved Algorithmic Complexity for the 3SEQ Recombination Detection Algorithm. *Mol Biol Evol* 2018; **35**(1): 247-51.

26. Minh BQ, Schmidt HA, Chernomor O, et al. IQ-TREE 2: New Models and Efficient Methods for Phylogenetic Inference in the Genomic Era. *Mol Biol Evol* 2020; **37**(5): 1530-4.

27. Rambaut A, Lam TT, Max Carvalho L, Pybus OG. Exploring the temporal structure of heterochronous sequences using TempEst (formerly Path-O-Gen). *Virus evolution* 2016; **2**(1): vew007.

28. Baele G, Ji X, Hassler GW, et al. BEAST X for Bayesian phylogenetic, phylogeographic and phylodynamic inference. *Nat Methods* 2025.

29. Bhatt S, Gething PW, Brady OJ, et al. The global distribution and burden of dengue. *Nature* 2013; **496**(7446): 504-7.

30. Layan M, Müller NF, Dellicour S, et al. Impact and mitigation of sampling bias to determine viral spread: Evaluating discrete phylogeography through CTMC modeling and structured coalescent model approximations. *Virus evolution* 2023; **9**(1): vead010.

31. Sagulenko P, Puller V, Neher RA. TreeTime: Maximum-likelihood phylodynamic analysis. *Virus evolution* 2018; **4**(1): vex042.

32. Ayres DL, Darling A, Zwickl DJ, et al. BEAGLE: an application programming interface and high-performance computing library for statistical phylogenetics. *Systematic biology* 2012; **61**(1): 170-3.

33. Ji X, Zhang Z, Holbrook A, et al. Gradients Do Grow on Trees: A Linear-Time O(N)-Dimensional Gradient for Statistical Phylogenetics. *Molecular Biology and Evolution* 2020; **37**(10): 3047-60.

34. Baele G, Gill MS, Lemey P, Suchard MA. Hamiltonian Monte Carlo sampling to estimate past population dynamics using the skygrid coalescent model in a Bayesian phylogenetics framework. *Wellcome open research* 2020; **5**: 53.

35. Taylor-Salmon E, Hill V, Paul LM, et al. Travel surveillance uncovers dengue virus dynamics and introductions in the Caribbean. *Nature communications* 2024; **15**(1): 3508.

36. Rambaut A, Drummond AJ, Xie D, Baele G, Suchard MA. Posterior Summarization in Bayesian Phylogenetics Using Tracer 1.7. *Systematic biology* 2018; **67**(5): 901-4.

37. Virk RK, Jayakumar J, Mendenhall IH, et al. Divergent evolutionary trajectories of influenza B viruses underlie their contemporaneous epidemic activity. *Proceedings of the National Academy of Sciences of the United States of America* 2020; **117**(1): 619-28.

38. Lemey P, Ruktanonchai N, Hong SL, et al. Untangling introductions and persistence in COVID-19 resurgence in Europe. *Nature* 2021; **595**(7869): 713-7.

39. Bahl J, Nelson MI, Chan KH, et al. Temporally structured metapopulation dynamics and persistence of influenza A H3N2 virus in humans. *Proceedings of the National Academy of Sciences of the United States of America* 2011; **108**(48): 19359-64.

40. du Plessis L, McCrone JT, Zarebski AE, et al. Establishment and lineage dynamics of the SARS-CoV-2 epidemic in the UK. *Science* 2021; **371**(6530): 708-12.

41. Ni H, Cai X, Ren J, et al. Epidemiological characteristics and transmission dynamics of dengue fever in China. *Nature communications* 2024; **15**(1): 8060.

42. United States CDC. CDC Yellow Book Map. <https://www.healthmap.org/dengue/en/>.

43. Jentes ES, Lash RR, Johansson MA, et al. Evidence-based risk assessment and communication: a new global dengue-risk map for travellers and clinicians. *Journal of travel medicine* 2016; **23**(6).

44. Añez G, Heisey DA, Espina LM, Stramer SL, Rios M. Phylogenetic analysis of dengue virus types 1 and 4 circulating in Puerto Rico and Key West, Florida, during 2010 epidemics. *The American journal of tropical medicine and hygiene* 2012; **87**(3): 548-53.

45. Muñoz-Jordán JL, Santiago GA, Margolis H, Stark L. Genetic relatedness of dengue viruses in Key West, Florida, USA, 2009-2010. *Emerging infectious diseases* 2013; **19**(4): 652-4.

46. Bulterys PL, Solis D, Verghese M, et al. Diagnosis of Dengue in a returning traveler from Pakistan suspected of COVID-19, California, USA. *Diagnostic microbiology and infectious disease* 2021; **101**(4): 115517.

47. Klitting R, Piorkowski G, Rousset D, et al. Molecular epidemiology identifies the expansion of the DENV2 epidemic lineage from the French Caribbean Islands to French Guiana and mainland France, 2023 to 2024. *Euro surveillance* 2024; **29**(13).

48. Navero-Castillejos J, Benitez R, Torner N, et al. Molecular Characterization of Imported and Autochthonous Dengue in Northeastern Spain. *Viruses* 2021; **13**(10).

49. Camprubí-Ferrer D, Tomazatos A, Balerdi-Sarasola L, et al. Assessing viral metagenomics for the diagnosis of acute undifferentiated fever in returned travellers: a multicenter cohort study. *Journal of travel medicine* 2024; **31**(3).

50. Navero-Castillejos J, Sánchez-Montalvá A, Sulleiro E, et al. Molecular Epidemiology of Travel-Associated and Locally Acquired Dengue Virus Infections in Catalonia, Spain, 2019. *Viruses* 2025; **17**(5).

51. Carletti F, Carli G, Spezia PG, et al. Genetic and structural characterization of dengue virus involved in the 2023 autochthonous outbreaks in central Italy. *Emerging microbes & infections* 2024; **13**(1): 2420734.

52. Lazzarini L, Barzon L, Foglia F, et al. First autochthonous dengue outbreak in Italy, August 2020. *Euro surveillance* 2020; **25**(36).

53. Shihada S, Emmerich P, Thomé-Bolduan C, et al. Genetic Diversity and New Lineages of Dengue Virus Serotypes 3 and 4 in Returning Travelers, Germany, 2006-2015. *Emerging infectious diseases* 2017; **23**(2): 272-5.

54. Yao MX, Wu SZ, Wang GL, et al. Imported dengue serotype 1 outbreak in a non-endemic region, China, 2017: A molecular and seroepidemiological study. *The Journal of infection* 2020; **81**(2): 304-10.

55. Cai L, Zhang H, He F, et al. Epidemiological and virus molecular characterization of dengue fever outbreak in Hunan province, 2018 [In Chinese]. *Chinese Journal of Epidemiology* 2020; **41**(12): 2119-24.

56. Guan J, He Z, Qin M, et al. Molecular characterization of the viral structural protein genes in the first outbreak of dengue virus type 2 in Hunan Province, inland China in 2018. *BMC infectious diseases* 2021; **21**(1): 166.

57. Qin Y, Zhang N, Zhen Q, et al. Analysis of the viral molecular characteristics in a dengue fever outbreak in Jiangsu province in 2023 [In Chinese]. *Chinese Journal of Experimental and Clinical Virology* 2025; **39**(1): 81-5.

58. Wang W, Yu B, Lin XD, et al. Reemergence and Autochthonous Transmission of Dengue Virus, Eastern China, 2014. *Emerging infectious diseases* 2015; **21**(9): 1670-3.

59. Song J, Ma J, Cui E, Huo Y. Epidemiological and genotypic analysis of 22 imported dengue fever cases in Henan Province [In Chinese]. *Modern Disease Control and Prevention* 2024; **35**(9): 709-13,17.

60. Xu G, Zhang Q, Zhang Y, et al. The first outbreak of dengue fever and molecular tracing in Puyang, 2019 [In Chinese]. *Chinese Journal of Preventive Medicine* 2021; **55**(8): 978-82.

61. Ma H, Du Y, Huang X, Li X, Xu B. Analysis of the genome sequences of Dengue virus caused an outbreak of Dengue Fever in Henan province, 2013 [In Chinese]. *Chinese Journal of Epidemiology* 2015; **36**(10): 1185-6.

62. Li D, Ma H, Li Y, et al. Analysis of the local dengue fever outbreak in the north of Henan Province in 2019 [In Chinese]. *Chinese Journal of Microbiology and Immunology* 2021; **41**(12): 948-53.

63. Wang W, Mou J, Wang H, Fang F, Zhu Y, Teng Z. Molecular characteristics of the whole genomes of DENV-1 strains isolated from dengue fever cases in Shanghai, China, from 2018 to 2020 [In Chinese]. *Chinese Journal of Zoonoses* 2022; **38**(6): 507-14.

64. Ma Y, Li S, Wan Z, et al. Phylogenetic analyses of dengue virus serotypes imported to Shanghai, China. *Journal of travel medicine* 2020; **27**(7).

65. Feng Y, Lin S, Pan M, Cao Y, Li W. Complete genome characterization and source tracking of Dengue virus from the first local dengue outbreak in Sichuan province, China [In Chinese]. *Chinese Journal of Vector Biology and Control* 2022; **33**(2): 239-44.

66. Li W, Jiang M, Zhong H, Cao Y, Feng Y. Molecular characteristics analysis of imported dengue virus E gene in Sichuan province, China, 2012-2021 [In Chinese]. *Chinese Journal of Experimental and Clinical Virology* 2022; **36**(5): 586-90.

67. Hong WX, Zhao H, Deng YQ, et al. Severe dengue due to secondary DENV-1 infection in Mainland China. *Journal of clinical virology : the official publication of the Pan American Society for Clinical Virology* 2013; **57**(2): 184-6.

68. Sang S, Liu Q, Guo X, et al. The epidemiological characteristics of dengue in high-risk areas of China, 2013-2016. *PLoS neglected tropical diseases* 2021; **15**(12): e0009970.

69. Jiang L, Su W, Liu W, Cao Y, Di B, Zhang Z. Epidemiological characteristics of dengue fever and molecular biological characteristics of envelope gene of dengue virus in Guangzhou, Guangdong, 2015−2019 [In Chinese]. *Disease Surveillance* 2024; **39**(9): 1135-42.

70. Jing QL, Yang ZC, Luo L, et al. Emergence of dengue virus 4 genotype II in Guangzhou, China, 2010: survey and molecular epidemiology of one community outbreak. *BMC infectious diseases* 2012; **12**: 87.

71. Guo C, Zhong X, Lin J, Zhang X, Zhang Z, Chen W. Comparative analysis of molecular characteristics of dengue virus E gene in Shantou city between 2018 and 2019 [In Chinese]. *Chinese Journal of Microbiology and Immunology* 2021; **41**(4): 295-300.

72. Li G, Pan P, He Q, et al. Molecular epidemiology demonstrates that imported and local strains circulated during the 2014 dengue outbreak in Guangzhou, China. *Virologica Sinica* 2017; **32**(1): 63-72.

73. Su W, Jiang L, Lu W, et al. A Serotype-Specific and Multiplex PCR Method for Whole-Genome Sequencing of Dengue Virus Directly from Clinical Samples. *Microbiology spectrum* 2022; **10**(5): e0121022.

74. Lin YP, Luo Y, Chen Y, et al. Clinical and epidemiological features of the 2014 large-scale dengue outbreak in Guangzhou city, China. *BMC infectious diseases* 2016; **16**: 102.

75. JIANG L-y, LIU Y, SU W-z, et al. Epidemiology of dengue virus serotype 4 cases and E gene analysis of dengue virus in Guangzhou Province from 2010 to 2019 [In Chinese]. *Chinese Journal of Disease Control & Prevention* 2021; **25**(11): 1360-4.

76. Sun J, Wu D, Zhou H, et al. The epidemiological characteristics and genetic diversity of dengue virus during the third largest historical outbreak of dengue in Guangdong, China, in 2014. *The Journal of infection* 2016; **72**(1): 80-90.

77. Yu J, Li X, He X, et al. Epidemiological and Evolutionary Analysis of Dengue-1 Virus Detected in Guangdong during 2014: Recycling of Old and Formation of New Lineages. *The American journal of tropical medicine and hygiene* 2019; **101**(4): 870-83.

78. SU W-z, CHEN Z-q, JIANG L-y, et al. Full-length sequencing and the genetic characteristics of imported dengue virus serotype Ⅲ isolates in Guangzhou in 2018 [In Chinese]. *Modern Preventive Medicine* 2020; **47**(6): 1082-6.

79. Bai Z, Liu LC, Jiang LY, et al. Complete genome sequence of dengue virus serotype 3 from guangzhou, china. *Genome announcements* 2013; **1**(2): e0020812.

80. Guo X, Zhao Q, Wu C, et al. First isolation of dengue virus from Lao PDR in a Chinese traveler. *Virology journal* 2013; **10**: 70.

81. Meng JX, Hu QM, Zhang LM, et al. Isolation and Genetic Evolution of Dengue Virus from the 2019 Outbreak in Xishuangbanna, Yunnan Province, China. *Vector borne and zoonotic diseases (Larchmont, NY)* 2023; **23**(6): 331-40.

82. Hu TS, Zhang HL, Feng Y, et al. Epidemiological and molecular characteristics of emergent dengue virus in Yunnan Province near the China-Myanmar-Laos border, 2013-2015. *BMC infectious diseases* 2017; **17**(1): 331.

83. Zhang J-y, Li L-h, Chen R, et al. Analysis of genotypic characteristics of dengue viruses in Jinghong, Yunnan Province, China, 2023 [In Chinese]. *Chinese Journal of Vector Biology and Control* 2025; **36**(2): 148-52.

84. Guo X, Yang H, Wu C, et al. Molecular Characterization and Viral Origin of the First Dengue Outbreak in Xishuangbanna, Yunnan Province, China, 2013. *The American journal of tropical medicine and hygiene* 2015; **93**(2): 390-3.

85. Zhang J, Shu Y, Shan X, et al. Co-circulation of three dengue virus serotypes led to a severe dengue outbreak in Xishuangbanna, a border area of China, Myanmar, and Laos, in 2019. *International journal of infectious diseases* 2021; **107**: 15-7.

86. Zhao Y, Li L, Ma D, et al. Molecular Characterization and Viral Origin of the 2015 Dengue Outbreak in Xishuangbanna, Yunnan, China. *Scientific reports* 2016; **6**: 34444.

87. Wang B, Yang H, Feng Y, et al. The distinct distribution and phylogenetic characteristics of dengue virus serotypes/genotypes during the 2013 outbreak in Yunnan, China: Phylogenetic characteristics of 2013 dengue outbreak in Yunnan, China. *Infection, genetics and evolution : journal of molecular epidemiology and evolutionary genetics in infectious diseases* 2016; **37**: 1-7.

88. Wang B, Li Y, Feng Y, et al. Phylogenetic analysis of dengue virus reveals the high relatedness between imported and local strains during the 2013 dengue outbreak in Yunnan, China: a retrospective analysis. *BMC infectious diseases* 2015; **15**: 142.

89. Guo X, Yang M, Jiang J, et al. Molecular characteristics of dengue virus outbreak in China-Myanmar border region, Yunnan province, 2015 [In Chinese]. *Chinese Journal of Epidemiology* 2016; **37**(3): 398-401.

90. Feng Y, Fan J, Zhu J, et al. Molecular epidemiology of an outbreak of Dengue fever in Jinghong city, Yunnan province, China, 2013 [In Chinese]. *Chinese Journal of Epidemiology* 2014; **35**(12): 1409-11.

91. YANG W-h, HUANG Y, FENG Y, CHEN H-y, ZHANG Y-z, ZHANG H-l. Investigation on dengue fever imported cases of Kunming city, Yunnan province in China and sequence analysis of C/PreM gene of dengue virus. *International Journal of Virology* 2015; **22**(1): 11-6.

92. Cao L, Yu Z, He H, et al. Retrospective investigation of the origin and epidemiology of the dengue outbreak in Yunnan, China from 2017 to 2018. *Frontiers in veterinary science* 2023; **10**: 1137392.

93. Lan Q, Shu Y, Li L, et al. Molecular characterization of structural protein genes of dengue virus serotype 1 epidemic in Yunnan, Southwest China, in 2018. *Arch Virol* 2021; **166**(3): 863-70.

94. Mo L, Shi J, Guo X, et al. Molecular characterization of an imported dengue virus serotype 4 isolate from Thailand. *Arch Virol* 2018; **163**(10): 2903-6.

95. Wen S, Ma D, Lin Y, et al. Complete Genome Characterization of the 2017 Dengue Outbreak in Xishuangbanna, a Border City of China, Burma and Laos. *Frontiers in cellular and infection microbiology* 2018; **8**: 148.

96. Wang B, Liang Y, Yang S, et al. Co-Circulation of 4 Dengue Virus Serotypes among Travelers Entering China from Myanmar, 2017. *Emerging infectious diseases* 2018; **24**(9): 1756-8.

97. Yu T-t, Kan N-p, Lin Q, Wang J-z. Epidemiological characteristics of dengue fever and molecular characteristics of indigenous epidemic strains in Fujian from 2019 to 2022 [In Chinese]. *Chinese journal of Zoonoses* 2025; **41**(4): 427-33.

98. Wang J, Chen H, Huang M, et al. Epidemiological and etiological investigation of dengue fever in the Fujian province of China during 2004-2014. *Science China Life sciences* 2017; **60**(1): 72-80.

99. Gao B, Zhang J, Wang Y, Chen F, Zheng C, Xie L. Genomic Characterization of Travel-Associated Dengue Viruses Isolated from the Entry-Exit Ports in Fujian Province, China, 2013-2015. *Japanese journal of infectious diseases* 2017; **70**(5): 554-8.

100. Wang JZ, You LB, Kan NP, Lin Q, Weng YW, Zheng KC. Frequent Import and Multiple Sources of Dengue Fever have Changed the Epidemic Situation of the Disease in Fujian Province, China. *Biomedical and environmental sciences : BES* 2020; **33**(2): 123-32.

101. Liu L, Wu T, Liu B, et al. The Origin and Molecular Epidemiology of Dengue Fever in Hainan Province, China, 2019. *Frontiers in microbiology* 2021; **12**: 657966.

102. Chen M, Tan Y, Tang Z, et al. Study of epidemiological characteristics and viral sources of dengue fever outbreak in Guangxi Zhuang Autonomous Region, 2014 [In Chinese]. *Chinese Journal of Epidemiology* 2016; **37**(10): 1350-5.

103. Wang J, F. B, Chen H, Ju Y, Kang N. E gene characterization of seven local infections of dengue virus in Guangxi in 2019 [In Chinese]. *Modern Preventive Medicine* 2022; **49**(18): 3421-5,35.

104. Sun H, Yao W, Siddique A, He F, Yue M. Genomic characterization of dengue virus serotype 2 during dengue outbreak and endemics in Hangzhou, Zhejiang (2017-2019). *Frontiers in microbiology* 2023; **14**: 1245416.

105. Yu H, Kong Q, Wang J, et al. Multiple Lineages of Dengue Virus Serotype 2 Cosmopolitan Genotype Caused a Local Dengue Outbreak in Hangzhou, Zhejiang Province, China, in 2017. *Scientific reports* 2019; **9**(1): 7345.

106. Yan H, Ding Z, Yan J, et al. Epidemiological Characterization of the 2017 Dengue Outbreak in Zhejiang, China and Molecular Characterization of the Viruses. *Frontiers in cellular and infection microbiology* 2018; **8**: 216.

107. Yi B, Chen Y, Ma X, et al. Incidence dynamics and investigation of key interventions in a dengue outbreak in Ningbo City, China. *PLoS neglected tropical diseases* 2019; **13**(8): e0007659.

108. Han A, Sun B, Sun Z, et al. Molecular Characterization and Phylogenetic Analysis of the 2019 Dengue Outbreak in Wenzhou, China. *Frontiers in cellular and infection microbiology* 2022; **12**: 829380.

109. Tittarelli E, Lusso SB, Goya S, et al. Dengue Virus 1 Outbreak in Buenos Aires, Argentina, 2016. *Emerging infectious diseases* 2017; **23**(10): 1684-5.

110. Tittarelli E, Mistchenko AS, Barrero PR. Dengue virus 1 in Buenos Aires from 1999 to 2010: towards local spread. *PloS one* 2014; **9**(10): e111017.

111. Moore PR, van den Hurk AF, Mackenzie JS, Pyke AT. Dengue viruses in Papua New Guinea: evidence of endemicity and phylogenetic variation, including the evolution of new genetic lineages. *Emerging microbes & infections* 2017; **6**(12): e114.
